# TGFβ signaling limits lineage plasticity in prostate cancer

**DOI:** 10.1371/journal.pgen.1007409

**Published:** 2018-05-21

**Authors:** Yi Hao, Glen A. Bjerke, Karolina Pietrzak, Tiffany A. Melhuish, Yu Han, Stephen D. Turner, Henry F. Frierson, David Wotton

**Affiliations:** 1 Department of Biochemistry and Molecular Genetics and Center for Cell Signaling, University of Virginia, Charlottesville, United States of America; 2 Department of Cytobiochemistry, University of Lodz, Lodz, Poland; 3 Department of Public Health Sciences, University of Virginia, Charlottesville, United States of America; 4 Department of Pathology, University of Virginia, Charlottesville, United States of America; Johns Hopkins University, UNITED STATES

## Abstract

Although treatment options for localized prostate cancer (CaP) are initially effective, the five-year survival for metastatic CaP is below 30%. Mutation or deletion of the *PTEN* tumor suppressor is a frequent event in metastatic CaP, and inactivation of the transforming growth factor (TGF) ß signaling pathway is associated with more advanced disease. We previously demonstrated that mouse models of CaP based on inactivation of Pten and the TGFß type II receptor (Tgfbr2) rapidly become invasive and metastatic. Here we show that mouse prostate tumors lacking Pten and Tgfbr2 have higher expression of stem cell markers and genes indicative of basal epithelial cells, and that basal cell proliferation is increased compared to Pten mutants. To better model the primarily luminal phenotype of human CaP we mutated Pten and Tgfbr2 specifically in luminal cells, and found that these tumors also progress to invasive and metastatic cancer. Accompanying the transition to invasive cancer we observed de-differentiation of luminal tumor cells to an intermediate cell type with both basal and luminal markers, as well as differentiation to basal cells. Proliferation rates in these de-differentiated cells were lower than in either basal or luminal cells. However, de-differentiated cells account for the majority of cells in micro-metastases consistent with a preferential contribution to metastasis. We suggest that active TGFß signaling limits lineage plasticity in prostate luminal cells, and that de-differentiation of luminal tumor cells can drive progression to metastatic disease.

## Author summary

Prostate cancer is among the leading causes of cancer deaths in men. While treatments for localized disease are quite effective, once the cancer metastasizes five-year survival rates drop to below 30%. The transforming growth factor (TGF) ß pathway is frequently inactivated in prostate cancer, and reduced expression of TGFß pathway components is associated with more advanced disease. Mouse models have shown that deletion of TGFß pathway components, in the background of a tumor initiating mutation, accelerates progression to invasive and metastatic prostate cancer. We analyzed the outcome of combining a deletion of the TGFß type 2 receptor (Tgfbr2) with deletion of the Pten tumor suppressor gene, which initiates tumorigenesis in the mouse prostate. Deletion of Tgfbr2 results in increased expression of markers of stem cell function and basal cell markers, and increased basal cell proliferation. Since human prostate cancer has a primarily luminal cell phenotype, we deleted Pten and Tgfbr2 specifically in luminal cells. This results in progression to invasive and metastatic cancer that is not seen with deletion of Pten alone. Analysis of the cell types in these tumors reveals that Tgfbr2 mutant luminal tumor cells differentiate to an intermediate cell type that has both basal and luminal characteristics, and also to basal cells. Analysis of early metastatic lesions suggests that the de-differentiated intermediate cells may be the drivers of metastasis.

## Introduction

Prostate cancer (CaP) is the second-leading cause of cancer deaths in men [1], with more than 161,000 cases and nearly 27,000 deaths predicted in the US in 2017 (http://seer.cancer.gov/statfacts/html/prost.html). Although five-year survival rates for patients with localized disease are high, for patients with distant metastases five-year survival is below 30%. Patients initially respond favorably to therapies based on androgen depletion reflecting the androgen-dependence of these tumors. However, anti-androgen therapy becomes ineffective as prostate cancers progress to a castration resistant state [2].

Transforming growth factor (TGF) ß family ligands assemble a complex of type I and type II receptors, resulting in type I receptor activation [3–5]. The activated type I receptor phosphorylates Smad proteins, primarily Smad2 and Smad3 for TGFß. Phosphorylated Smads bind Smad4 and accumulate in the nucleus where they regulate target gene expression [6]. In many cell types, including epithelial cells, TGFβ signaling via Smad2 and Smad3 promotes a G1 cell cycle arrest preventing uncontrolled cell proliferation. Thus, TGFß signaling frequently plays a tumor suppressive role [7, 8]. The TGFβ signaling pathway is disrupted by mutation or loss of expression of pathway components in many human cancers, including CaP [9–11]. Reduced expression of the TGFβ type I and type II receptors (encoded by the *TGFBR1* and *TGFBR2* genes) is associated with increased Gleason score and decreased survival, and reduced SMAD4 expression is also found in advanced human CaP [9, 12–14].

Inactivation of the *PTEN* tumor suppressor is found in more than 30% of primary human prostate tumors and in approximately 60% of CaP metastases [15–18]. In mice prostate specific deletion of the *Pten* tumor suppressor is one of the more robust models of prostate cancer. All mice develop prostate intraepithelial neoplasia (PIN) by six weeks of age that rapidly progresses to high grade PIN (HGPIN). In this model, HGPIN eventually develops to locally invasive cancer, but this occurs relatively slowly, and metastases are rarely seen [19–23]. Combining additional mutations with Pten deletion accelerates the progression from HGPIN to invasive poorly differentiated adenocarcinoma (PDA) and results in metastasis. Deletion of either Smad4 or Tgfbr2, to inactivate TGFß signaling in prostate epithelial cells, drives invasion and metastasis in the background of a Pten deletion [20, 24]. Combining other oncogenic hits with a Pten deletion has also been shown to increase invasion and metastasis. For example expression of a mutant Kras together with Pten deletion resulted in metastasis to lung and liver [25]. Although inactivating mutations in the *APC* tumor suppressor gene are less frequent in human CaP than in colon cancer, for example, nuclear β-catenin is observed in the majority of cases of advanced human CaP suggesting that this pathway is frequently de-regulated [26]. More recent genomic analyses of human prostate cancer suggest that *APC* mutations are found at a higher frequency than originally thought, with these being primarily nonsense and frame-shift mutations [27]. In mouse models, deletion of the *Apc* gene in mouse prostate epithelium results in HGPIN [28], which rapidly progresses to invasive cancer when combined with loss of the *Tgfbr2* gene [19].

The two major epithelial cell types within the prostate are luminal and basal cells, and there is evidence for stem cell pools within both populations. Lineage tracing in mouse prostate suggests that multipotent progenitors present within the basal cell population may give rise to unipotent basal and luminal progenitors during post-natal development [29]. More recent work with organoids suggests that the luminal population also contains stem cells capable of regenerating both basal and luminal cell types, albeit at a much lower frequency than basal cells [30]. The majority of cells in human prostate cancer have a luminal phenotype, although rare basaloid carcinomas are seen [31]. This is consistent with the idea that luminal cells are the cell of origin for most of human CaP. Prostate luminal cells have been shown to have the potential to be cancer initiating cells, and it has been suggested that castration resistant cells within this population may be the tumor initiating cells [32–34]. However, a number of studies have also suggested that basal cells can be a cell of origin for prostate cancer [32, 35, 36], and a basal-like stem cell gene expression signature is associated with aggressive cancer [37]. Despite the differing requirements for the androgen receptor (AR) in basal and luminal cell survival, loss of Pten can overcome the effect of AR deletion, allowing luminal-like tumors to develop from both cell types [38]. Deletion of Pten may limit androgen-responsive gene expression by modulating AR function, decreasing the ability of the AR to promote differentiation [39]. Recent work has suggested that lineage plasticity in human CaP may allow for a fraction of tumor cells to escape the effects of anti-androgen therapies, allowing the tumor to recur as a therapy resistant cancer despite initial regression of the primary tumor [40]. In mouse prostate, the deletion of Rb1 together with Pten results in increased lineage plasticity and metastasis with a gene expression signature reminiscent of human neuroendocrine CaP [41]. This may be one mechanism to explain the eventual failure of androgen ablation, suggesting that a better understanding of linage plasticity, rather than the initial cell of origin, is of importance.

Disruption of TGFß signaling increases proliferation and overcomes senescence in prostate tumors initiated by loss of either Pten or Apc, and accelerates progression to a widespread invasive phenotype [19, 20, 24]. To further understand the cellular changes induced by loss of Tgfbr2 expression in mouse prostate tumors we used transcriptional profiling of these two tumor models. This analysis reveals increased expression of genes associated with stem-like properties in these highly proliferative tumors. In addition we observed increased numbers of basal cells, which make up much of the invasive cancer, and we show that loss of Tgfbr2 primarily affects basal cell proliferation. However, deletion of Tgfbr2 and Pten specifically in luminal cells resulted in metastatic cancer and extensive de-differentiation to an intermediate cell type expressing both basal and luminal markers. Analysis of early lung metastatic lesions indicates that these de-differentiated prostatic cells may preferentially generate metastases, suggesting that increased lineage plasticity in the absence of TGFß signaling may contribute to an aggressive disease phenotype.

## Results

### Loss of Tgfbr2 causes large scale transcriptome changes

We have previously shown that deletion of *Tgfbr2* from mouse prostate epithelium accelerates tumor progression in the background of either a *Pten* or *Apc* null mutation [19, 20]. To examine the status of the TGFß signaling pathway in human CaP we analyzed a panel of high grade (primarily Gleason score 8–10) human tumors by IHC for SMAD4 and active phosphorylated SMAD2 (pSMAD2). We chose these as pSMAD2 provides a readout for receptor activity (the combination of both Type I and Type II), and SMAD4 is required to complex with the receptor activated SMAD. More than half of the 38 tumors analyzed had reduced expression of SMAD4 and a slightly higher number had low pSMAD2 signal (S1 Fig). These proportions are similar to that seen for activation of AKT in this set of samples, and is consistent with previous reports showing reduced expression of both Type I and Type II receptors, and of SMAD4 in human CaP [9–14, 24].

**Fig 1:**
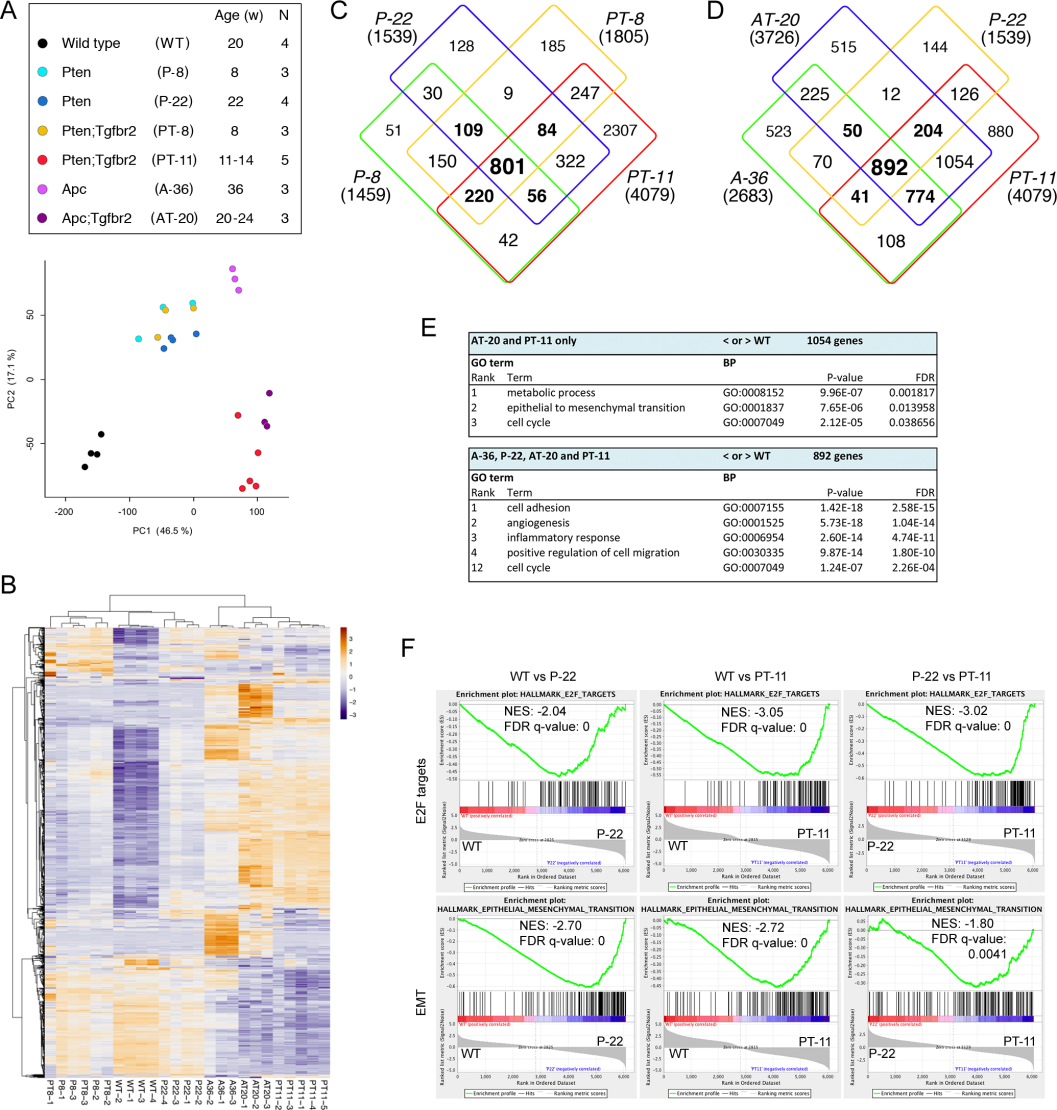
Transcriptome analysis of mouse prostate tumors. A) Principle component analysis of tumor RNA-seq data from mice of the indicated ages and genotypes. Names of the groups used in subsequent panels are shown in brackets. B) Unsupervised hierarchical clustering using the 1000 most variable genes of the RNA-seq data. C and D) Overlap in gene expression changes (log2 fold change > +/- 1.0, p adjusted < 0.0001) for each tumor group compared to wild type prostates. The numbers of genes in total are shown in brackets. E) GO analysis for biological process terms for genes that are differentially expressed in the two groups with invasive cancer compared to wild type, and for all four groups of tumors (excluding the 8-week sets) compared to wild type. F) GSEA analysis comparing 22-week Pten (P-22) to wild type, 11-week Pten;Tgfbr2 (PT-11) to wild type and PT-11 to P-22, showing enrichment for E2F target genes and an EMT signature.

To begin to examine how loss of TGFß signaling drives prostate tumor progression we performed transcriptome profiling on normal wild type prostate and on tumors from mice with prostate-specific deletion of Apc, or Pten, with or without deletion of Tgfbr2. For the Apc mutants, we isolated tumors at 36 weeks of age, when they had extensive adenosquamous HGPIN, and at 20–24 weeks from the Apc;Tgfbr2 mutants when they first showed adverse signs of tumor burden. For the Pten model, we analyzed two different ages each for both single (Pten) and double (Pten;Tgfbr2) mutants. Tumors were isolated from both groups at 8 weeks of age when the predominant phenotype in both is HGPIN, and at 22 weeks of age from the Pten single mutants, which still have primarily HGPIN. Since double mutants do not generally survive beyond ~15 weeks, we isolated tumors at 11–14 weeks when they first showed signs of excess tumor burden. We anticipated that this combination would provide a comparison of progression in the more relevant Pten based model as well as comparison to a second model in which the tumor-initiating event is different. As shown in Fig 1A, principle component analysis (PCA) separated the samples into three broad groups, with the wild type samples clustering tightly together. The other two groups primarily appeared to cluster by phenotype, with less separation between the Pten and Apc based tumors. Thus, the eight-week Pten;Tgfbr2 double mutants clustered with the 8- and 22-week Pten single mutants, all of which have HGPIN as the primary phenotype. Unsupervised hierarchical clustering based on the 1000 most variable genes revealed a similar pattern (Fig 1B), with the older Pten;Tgfbr2 and Apc;Tgfbr2 tumors grouped together, although there are clearly some differences between them. Again, eight-week tumors, whether Pten single mutant or Pten;Tgfbr2 double do not separate into distinct groups and also appear similar to the older Pten single mutants. To examine which genes are differentially expressed among the different groups, we performed all pairwise comparisons between the seven groups of samples, and identified genes as differentially expressed if there was a log2-fold difference of greater than +/-1.0, with an adjusted p-value of < 0.0001. From these comparisons, we first focused on differences between each group of tumors and the wild type prostates. Despite the relatively stringent cut-off these comparisons revealed large numbers of genes that were differentially expressed between wild type and each tumor group (S1A and S1B Table). For example, 4079 genes passed this cut-off when comparing the older Pten;Tgfbr2 tumors to wild type, with somewhat lower numbers from the comparisons of other groups to the wild type (Fig 1C and 1D). Comparison of the 8-week Pten;Tgfbr2 tumors and Pten single mutants at both ages revealed largely overlapping gene expression changes, although changes in these samples were quite variable, possibly due to the mix of normal and tumor tissue at this early age. In the older Pten;Tgfbr2 tumors a large fraction of the changes in gene expression were not seen in these other three groups (Fig 1C). Given the lack of difference between the two 8-week groups, and the variability among these early tumors we focused on the older Pten single and double mutants, and compared them to the Apc and Apc;Tgfbr2 mutant tumors. Almost 900 genes were differentially expressed in all four tumor types compared to wild type, with a slightly larger number shared only between the two double mutant tumors (Fig 1D). GO analysis revealed that two of the top three enriched biological process terms in the genes changing in only the two double mutants were epithelial to mesenchymal transition (EMT) and cell cycle, consistent with the double mutants being more proliferative and invasive (Fig 1E, S1C Table). GSEA analysis comparing wild type to 22-week Pten and to 11-week Pten;Tgfbr2 mutants, and comparing these two groups of tumors to each other showed increasing enrichment for E2F targets with progression to the more aggressive tumors, but suggested that the EMT signature was already present in the Pten single mutant tumors (Fig 1F).

### Increased expression of genes with stem cell function in invasive tumors

**Fig 2:**
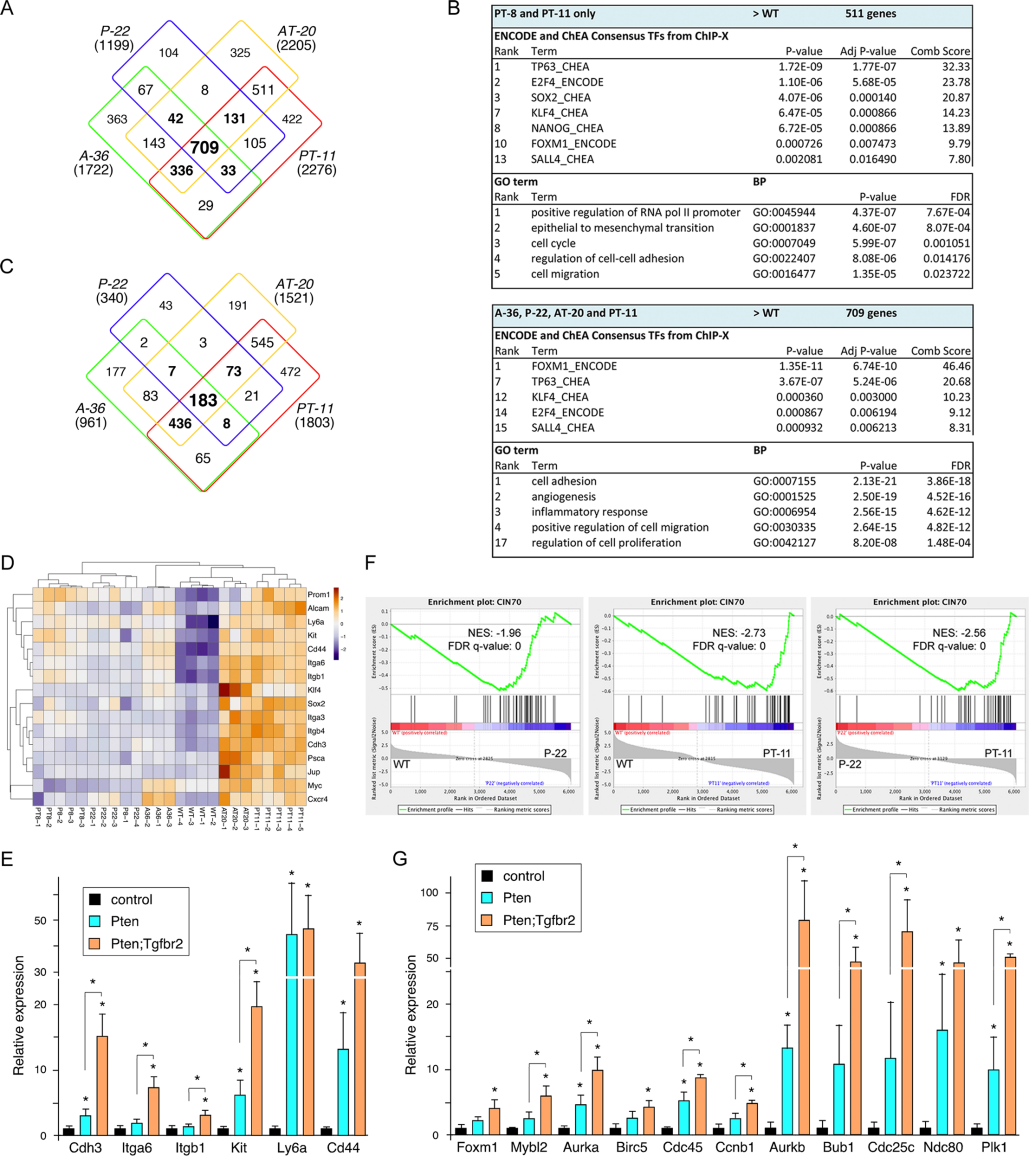
Expression of genes associated with proliferation and stem like function. A) Overlap between genes with increased expression (log2 fold change > +1.0, p adjusted < 0.0001) in tumors compared to wild type for each of the four tumor groups shown. B) Enrichment analysis for putative transcription factor binding and GO biological process terms in genes with increased expression in double mutant tumors or in all four groups. C) Overlap between genes with lower expression (log2 fold change < -1.0, p adjusted < 0.0001) in tumors compared to wild type for each of the four tumor groups shown. D) Heat-map showing relative changes in expression for a selection of genes with links to stem cell-like function in cancer. E) A selection of the gene expression changes from D were validated by qRT-PCR in Pten and Pten;Tgfbr2 tumors (N = 4 each) shown as mean + sd. F) GSEA analysis of the CIN70 dataset that is associated with more aggressive tumors. G) qRT-PCR validation in Pten and Pten;Tgfbr2 tumors of a selection of genes from the CIN70 set and from the other GSEA shown in S1 Fig. * p < 0.05 by student’s T test.

The previous analyses suggest that with the transition from HGPIN to invasive cancer there is an enrichment for gene changes associated with cell cycle progression and an underlying enrichment for EMT in the HGPIN tumors. Comparison of increases in gene expression relative to the wild type samples revealed a similar pattern to that seen with all gene expression changes, and enrichment for GO terms associated with cell cycle, EMT and regulation of transcription (Fig 2A and 2B and S1C Table). In contrast, many fewer genes were consistently decreased in expression compared to the wild type, and GO analysis did not reveal significant enrichment of any terms among these groups (Fig 2C). Analysis of ENCODE and ChEA transcription factor binding data suggested an enrichment for genes bound by transcription factors associated with stem cell function, such as SOX2, KLF4, NANOG and SALL4 among genes with increased expression in the tumors compared to wild type (Fig 2B). Scanning the RNA-seq data we noticed that a number of markers associated with stem cell-like function in cancer were increased in the double mutant tumors compared to wild type and to the single mutants (Fig 2D). To validate these changes, we focused on the wild type, Pten single and Pten;Tgfbr2 double mutant tumors, since the Pten null model is more relevant to human CaP. Analysis of a distinct set of tumors of these genotypes by qRT-PCR demonstrated increased expression of a panel of cancer stem cell markers in the Pten single mutants compared to wild type, with further increases in the double mutant tumors (Fig 2E). Several gene sets that are associated with more aggressive tumors or with stem-like features of human tumors have been identified. For example, the CIN70 gene set, which is associated with more aggressive tumors that have higher levels of chromosome instability includes a number of genes with mitotic functions, including FOXM1 [42]. This gene set may also be an indication of highly proliferative tumors. GSEA analysis with the CIN70 set revealed increasing enrichment in Pten null and Pten;Tgfbr2 double null tumors (Fig 2F). Since FOXM1 targets were also enriched in the tumor samples based on ENCODE ChIP-seq data (Fig 2B), we also compared to a FOXM1 target gene set identified by ChIP-seq [43]. This gene set showed enrichment in the tumor samples, and further enrichment comparing double null to single null tumors (S2 Fig). Similar patterns were seen with other data-sets, including an embryonic stem cell (ESC) associated signature identified in a pan cancer analysis as being enriched in aggressive tumors [44] (S2 Fig). Although there is some overlap amongst the genes in these three data-sets, there are also large numbers of genes that are distinct to each, particularly when comparing the ESC and FOXM1 data-sets (S2B Fig). To confirm the changes seen in the RNA-seq data, we again performed qRT-PCR on wild type prostates and Pten single and double null tumors, probing a selection of the genes present in these three data sets. The majority of genes tested were increased in the Pten single null tumors and most increased when comparing double to single mutant tumors (Fig 2G). In this analysis we examined both Foxm1 and Mybl2, as these two transcription factors have been shown to co-regulate a number of the mitotic target genes that are present in these data sets. Both showed increased expression in the double mutant tumors, and similar patterns were seen with known target genes, such as Ccnb1. Together these analyses suggest that loss of TGFß signaling in the background of a Pten null prostate tumor further activates pro-proliferative and stem-like gene expression programs, which may already be initiated by the loss of Pten.

### Increased basal cells in Tgfbr2 null tumors

**Fig 3:**
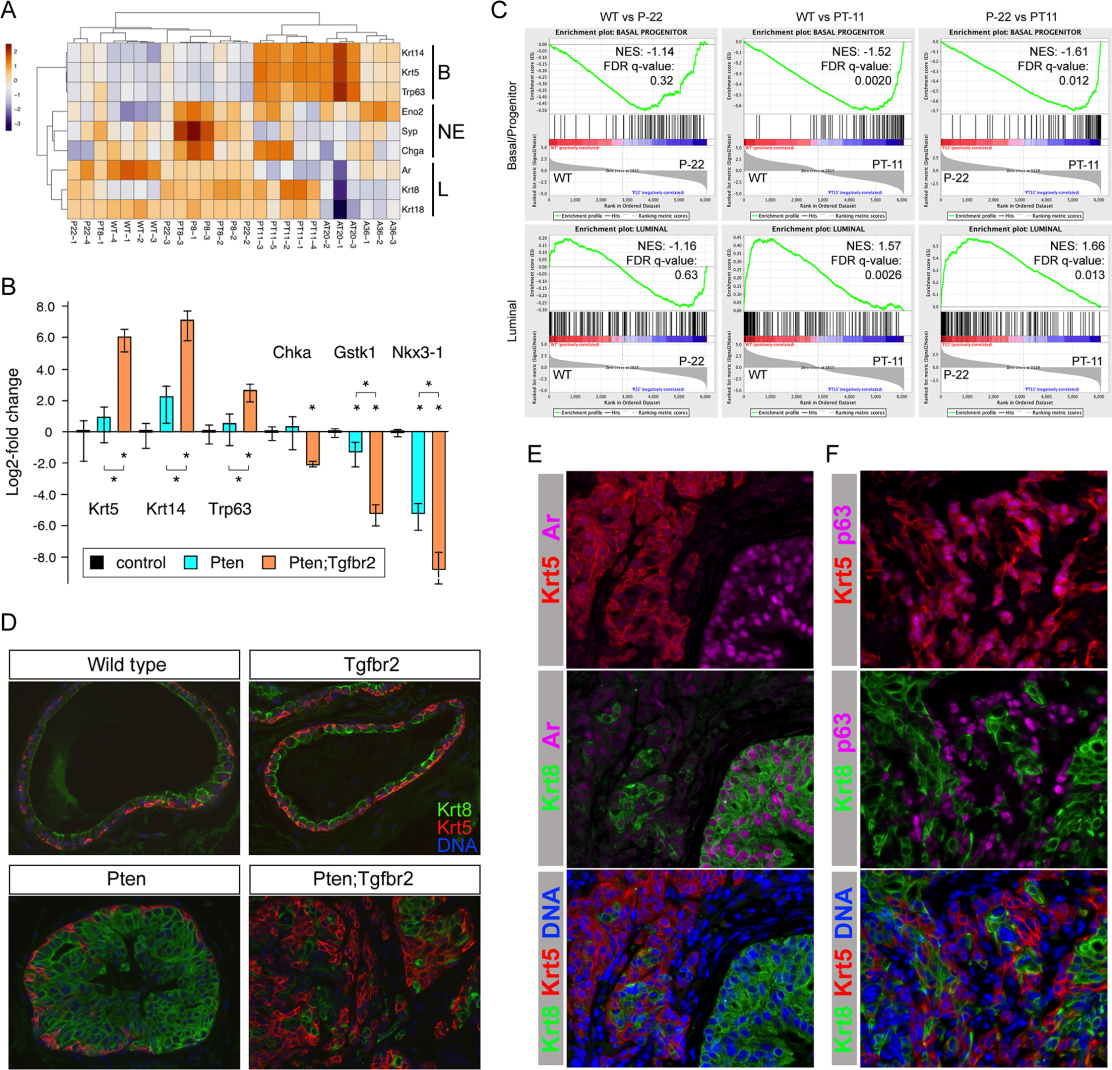
Increased basal cells in invasive cancer. A) Heat-map showing relative changes in expression for a small panel of genes indicative of luminal (L), basal (B) or neuroendocrine (NE) cell types. B) qRT-PCR analysis of three basal and three luminal enriched genes (plotted as log2-fold change) in Pten and Pten;Tgfbr2 tumors (N = 4 each) shown as mean + sd. * p < 0.05 by student’s T test. C) GSEA comparing P-22 to wild type, PT-11 to wild type and PT-11 to P-22 tumors, showing enrichment of basal/progenitor genes in the Pten;Tgfbr2 tumors and of a luminal signature in Pten tumors. D) IF analysis of prostate samples of the indicated genotypes for luminal (Krt8) and basal (Krt5) markers. Merged images only shown. E) Analysis of Krt5, Krt8 and Ar expression by IF. Merged images of Krt5 or Krt8 with Ar, and Krt5, Krt8 and DNA are shown. F) Analysis of Krt5, Krt8 and p63 expression by IF. Merged images of Krt5 or Krt8 with p63, and Krt5, Krt8 and DNA are shown. E and F from analysis of Pten;Tgfbr2 tumors.

Among the most highly expressed and significantly up-regulated genes in the 11-week old Pten;Tgfbr2 tumors were Krt5 and Krt14, two basal cell specific keratin genes. In addition, expression of the basal cell-enriched p53 paralog, Trp63, was significantly increased in Pten;Tgfbr2 tumors. Comparing expression of three genes each that are representative of basal, luminal or neuroendocrine cells showed clear increases in expression of the basal genes in the double null tumors (Fig 3A). Luminal specific genes were generally lower in the Apc-based tumors. There was some apparent increase in neuroendocrine genes in the 8-week tumors, but it should be noted that expression of these genes is very low and quite variable, and the changes did not reach statistical significance. Increased expression of Trp63 and the two basal keratins in Pten;Tgfbr2 tumors was validated by qRT-PCR, and for at least some luminal-enriched genes we observed a decrease in expression (Fig 3B). The basal cell population has been thought to contain stem cells that can regenerate the entire prostate, although recent evidence suggests that such stem cells may also reside in the luminal population [29, 30]. Analysis of luminal and basal/progenitor-like cell populations isolated from normal mouse prostate, based on expression of fluorescent marker transgenes, identified gene expression signatures associated with each [45]. Comparison of our RNA-seq data to these two data-sets revealed enrichment of the basal/progenitor gene set in the Pten;Tgfbr2 tumors and an enrichment of the luminal signature in Pten single null tumors when compared to the double nulls (Fig 3C). Normal prostate ducts in the mouse are primarily composed of a single layer of luminal epithelial cells with an incomplete basal layer surrounding it. Immunofluorescent detection of basal (Krt5) and luminal (Krt8) keratins showed that this organization is not altered in the absence of Tgfbr2 and, as previously reported, there is no significant phenotype associated with loss of Tgfbr2 alone (Fig 3D) [19, 20]. Where HGPIN was observed in the Pten single mutant tumors, the ducts become filled with luminal cells, a pattern that was also seen in HGPIN ducts in the double mutant tumors. However, in the Pten;Tgfbr2 tumors, a large proportion of the invasive cancer consisted of cells that were strongly positive for Krt5, with Krt8 cells intermixed (Fig 3D). To better characterize the cell types present in the Pten;Tgfbr2 tumors we examined expression of additional markers. The androgen receptor (AR) was highly expressed in luminal cells in intact ducts that were also strongly Krt8 positive. AR was present at a lower level in the Krt5 positive invasive cancer, and was clearly absent from stromal cells (Fig 3E). Trp63 expression was largely absent from cells that stained strongly for Krt8, but was clearly present in Krt5 positive cells, consistent with these being basal cells (Fig 3F). Thus deletion of Tgfbr2 in the context of a tumor initiating mutation results in an increase in the basal cell population together with a rapid transition to invasive cancer.

### Increased basal cell proliferation in invasive cancer

**Fig 4:**
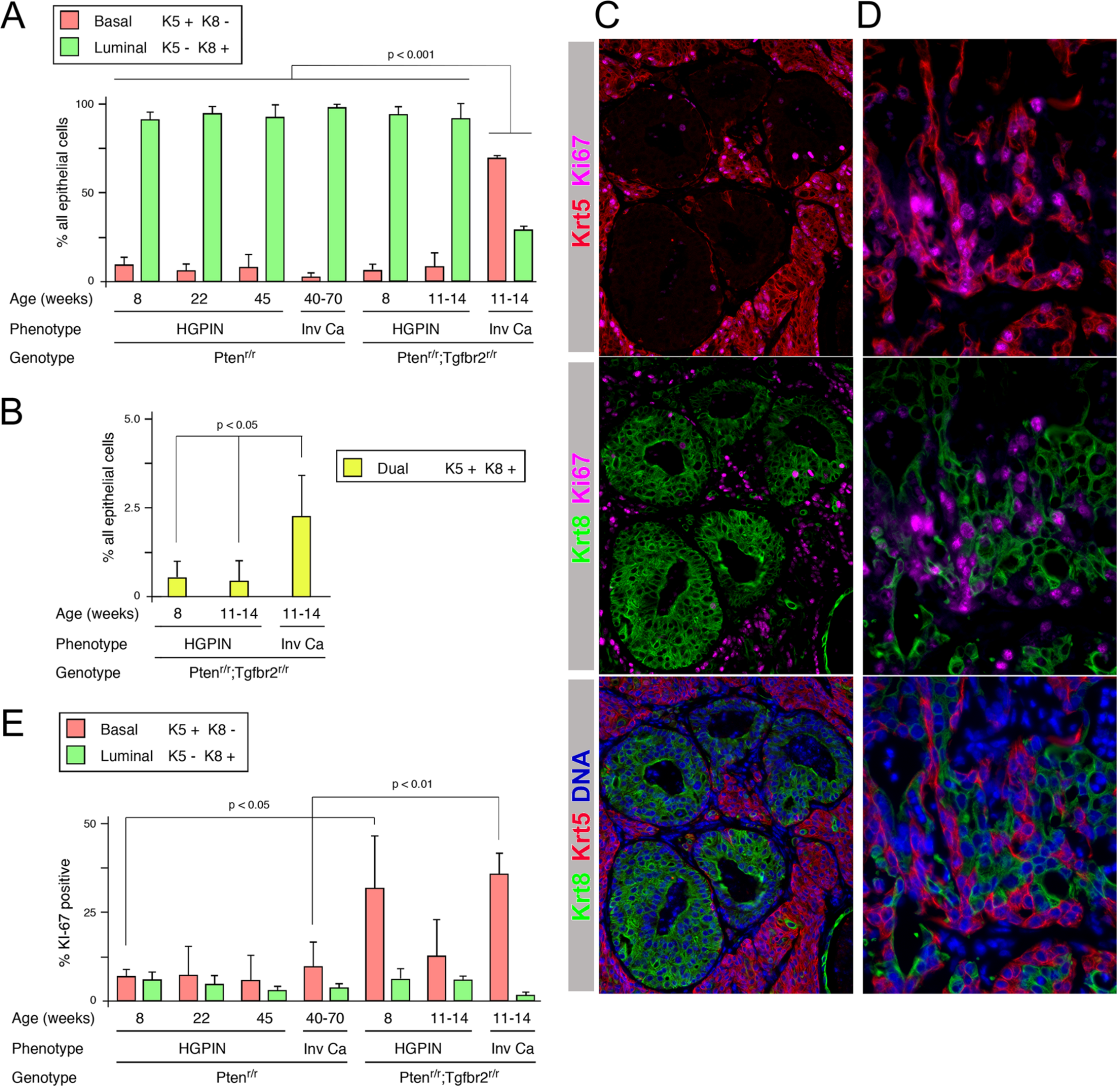
Increased basal cell proliferation in Tgfbr2 mutant tumors. A) A quantification of the proportion of basal and luminal cells in tumors of the indicated ages, genotypes and phenotypes. B) The percentage of Krt5/Krt8 dual positive cells is shown, as in A. C and D) representative images of Pten;Tgfbr2 tumors stained for Krt5, Krt8 and Ki67 (and with Hoechst 33342 for DNA) are shown (Krt5 plus Ki67 –top, Krt8 plus Ki67 –center, Krt5, Krt8 and DNA–bottom). C shows a region with both invasive cancer and HGPIN. D shows invasive cancer at higher magnification. E) Quantification of the proportion of proliferating (Ki67 positive) basal and luminal cells in tumors of the indicated ages, genotypes and phenotypes (For A, B, E: N = 3–5 mice per group, >180 cells counted per mouse).

To better assess the increase in basal cell numbers in the Pten;Tgfbr2 tumors we stained a series of Pten and Pten;Tgfbr2 null samples for both Krt5 and Krt8 and quantified the proportion of cells in each. As shown in Fig 4A, in Pten null tumors HGPIN was composed of more than 90% luminal cells at all ages examined. Even in invasive cancers in older Pten null tumors, most of the invasive cells were luminal (Fig 4A and S3 Fig). Similarly, in Pten;Tgfbr2 tumors HGPIN was primarily luminal, with no significant difference in the proportion of basal cells compared to HGPIN in the Pten null. In contrast, in invasive regions in the Pten;Tgfbr2 tumors there were more than twice as many basal as luminal cells (Fig 4A). While performing this analysis we also examined the number of cells that were positive for both basal and luminal keratins. These cells were extremely rare in HGPIN in Pten;Tgfbr2 tumors, but were slightly increased in the invasive cancer (Fig 4B). However, it should be noted that even in the invasive cancer these dual positive cells were still only a very small fraction of the total.

Given the increased frequency of basal cells, we tested whether this was due to a specific increase in basal cell proliferation. We examined a panel of tumors for expression of both Krt5 and Krt8, together with Ki67 to identify actively proliferating cells within each population. In Pten;Tgfbr2 tumors, the invasive cancer was highly proliferative and was largely Krt5-high and Krt8-low, whereas fewer cells stained positive for Ki67 in intact HGPIN ducts that were luminal (Fig 4C). Closer examination of regions of invasive cancer in which both basal and luminal cells were present suggested that within the invasive cancer more Krt5-high cells were proliferating than Krt8-high cells (Fig 4D). When we quantified this across both genotypes and a range of ages and phenotypes, there was little difference between proliferative rates in basal and luminal cells in HGPIN, except in early Pten;Tgfbr2 tumors, where there was an increased rate in the basal cells compared to the luminal, although these numbers were quite variable (Fig 4E). The most striking difference was the high proliferation rate of basal cells in invasive cancer in the Pten;Tgfbr2 tumors compared to those in the Pten or in intact HGPIN ducts in the same animals, consistent with a preferential effect of TGFß signaling on basal cell proliferation. It is also possible that luminal to basal de-differentiation contributes to the increase in basal cells in the invasive tumors, although these data clearly show a role for increased basal cell proliferation.

As discussed before, the majority of human CaP exhibits a luminal phenotype. However, there has been some interest in basal and luminal subtyping of human CaP by the identification of gene expression signatures that correlate with disease progression. Analysis of human CaP data using a 50 gene set that had been shown to distinguish the four types of human breast cancer, identified three human CaP subtypes that matched the luminal A, luminal B and basal subtypes from this gene set [46, 47]. Comparison of our RNA-seq data with this gene set suggested a preferential enrichment for increased expression of genes associated with the luminal B subtype, rather than the basal signature, in the double null tumors (S4A Fig). Comparison to a different 37 gene signature, that again identified two luminal subtypes and one basal human CaP subtype [48], suggested increased expression of the genes indicative of the more aggressive of the two luminal signatures in the *Pten;Tgfbr2* double null tumors (S4B and S4C Fig). Thus, although there is a significant basal cell component to these tumors, they appear to contain links to gene expression signatures indicative of aggressive luminal tumors.

### Invasive prostate cancer with luminal specific deletion of Pten and Tgfbr2

**Fig 5:**
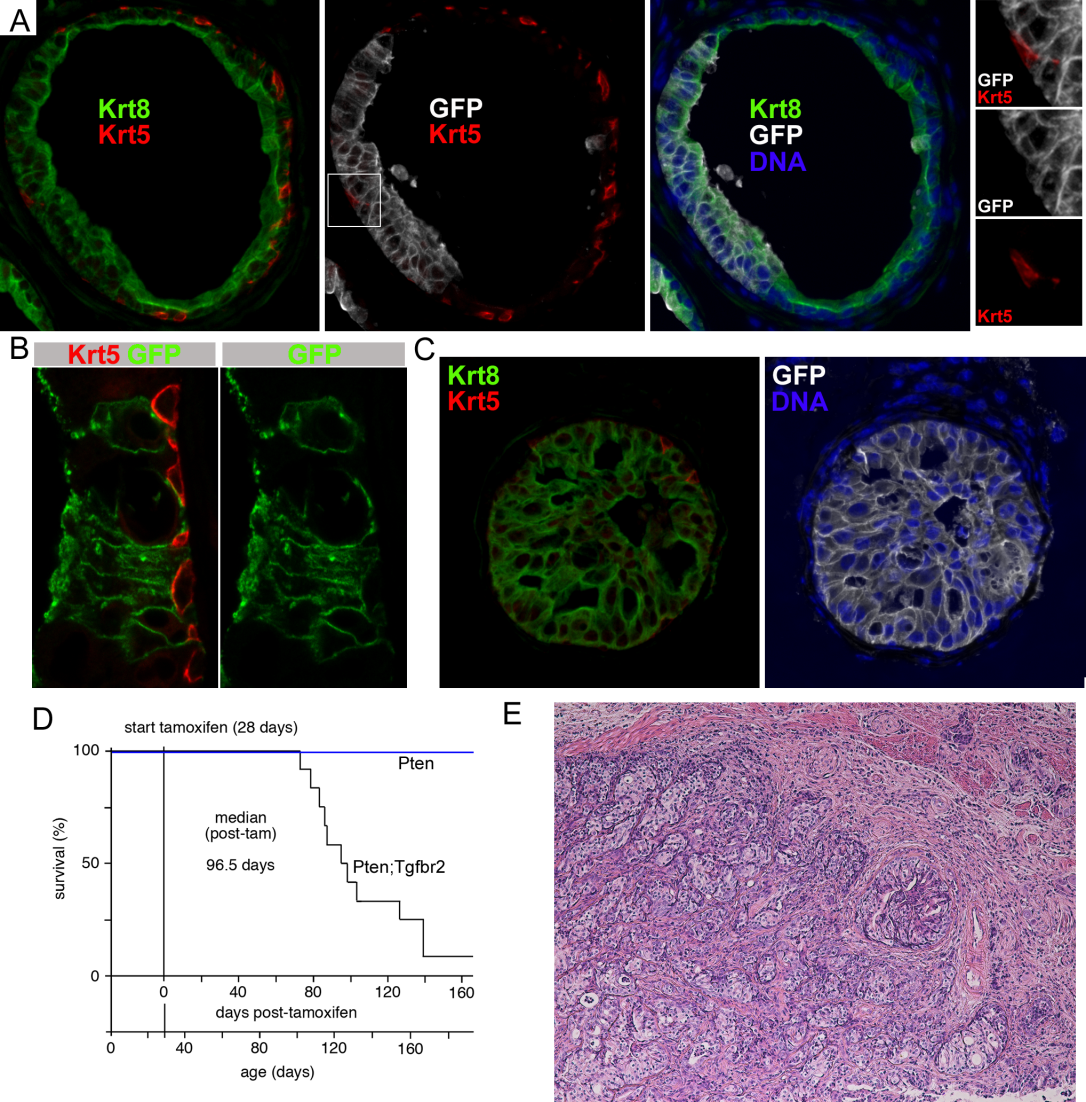
Luminal-specific deletion of Pten and Tgfbr2 causes invasive cancer. A) IF analysis of Krt5, Krt8, GFP and DNA in prostate from mice with a Krt8-CreERT2 transgene, a GFP recombination reporter and conditional Pten and Tgfbr2 alleles. Images are from 5 weeks after tamoxifen treatment. The boxed region in the center panel is shown at higher resolution on the right. B) Confocal imaging of Krt5 and GFP in prostate 4 weeks after tamoxifen. C) IF analysis for Krt5, Krt8, GFP and DNA in an HGPIN duct at 8 weeks after tamoxifen. D) Survival analysis of a cohort of 12 mice with conditional Pten and Tgfbr2 alleles and six Pten mice following initiation of tamoxifen treatment at 4 weeks of age. E) H&E staining of a region of invasive cancer in a Krt8-Cre Pten;Tgfbr2 tumor from a mouse that was euthanized for tumor burden at ~12 weeks post-tamoxifen.

Human prostate cancer cells typically have a luminal phenotype, whereas in the PbCre4 model the Cre is expressed in both basal and luminal cells and deletion of Tgfbr2 appears to activate basal cell proliferation. Therefore, we examined the effect of deleting Pten and Tgfbr2 specifically in luminal cells. To do this we combined the conditional alleles of Pten and Tgfbr2 with a lineage tracing marker (mTmG) and a tamoxifen inducible Krt8-Cre transgene [49, 50]. This combination allows for deletion specifically in luminal epithelial cells, and an ability to permanently track which cells have undergone Cre activation by examining expression of GFP following excision of the STOP cassette. To induce recombination driven by the Krt8-CreERT2 we initiated tamoxifen treatment by gavage at four weeks of age and examined tissues by IF detection of the GFP lineage tracing allele to determine whether there was significant recombination in the prostate. Co-staining for GFP together with Krt8 and Krt5 revealed a clear overlap between the GFP signal and Krt8, whereas basal cells lacked detectable GFP expression (Fig 5A). From examination of 59 GFP positive normal ducts and 115 ducts with focal PIN from six mice at 2–6 weeks post-tamoxifen we did not identify any clearly GFP positive basal cells. In some cases where GFP positive luminal cells were directly adjacent to a basal cell it may be difficult to exclude partial co-localization of the GFP and Krt5 signal. However, we never observed GFP signal close to a basal cell without adjacent luminal cells that were clearly GFP positive (see Fig 5A). Furthermore, by confocal analysis we were unable to detect any GFP signal that was clearly present in a basal (Krt5 positive) cell (Fig 5B and S5A Fig). Co-staining of prostates six weeks after tamoxifen for GFP and pAkt showed that Akt was activated in GFP positive cells, consistent with loss of Pten in these cells (S5B Fig). Similarly, Tgfbr2 expression was reduced in GFP positive cells compared to adjacent GFP negative cells (S5C Fig). Together with previous analyses that have used this transgene to drive luminal specific deletions [29], this suggests that if deletion occurs in cells other than luminal cells it is extremely rare.

To examine the initial phenotype resulting from deletion of Pten and Tgfbr2 in luminal cells we examined GFP expression at 5–6 weeks post-tamoxifen treatment. At this stage we observed a range of phenotypes from normal tissue to HGPIN, with all HGPIN ducts containing GFP positive cells. The proportion of GFP positive cells in ducts with HGPIN was significantly higher than in non-phenotypic ducts, with the mean proportion of GFP positive cells per duct being 25% at this stage, rising to almost 70% for HGPIN ducts (S6A Fig). Among 143 ducts examined 106 had GFP positive cells, with none of the GFP negative ducts exhibiting any phenotype, whereas, all HGPIN ducts were GFP positive (S6B and S6C Fig). In ducts with HGPIN, the GFP positive cells were strongly positive for Krt8, and basal cells at the edges of the ducts did not have GFP signal (Fig 5C). We next followed twelve Pten;Tgfbr2 Krt8-CreERT2 mice after tamoxifen treatment beginning at 4 weeks of age to examine survival of these mice. Of the twelve, eleven had to be euthanized for tumor burden by 20 weeks post-tamoxifen treatment. All eleven had large prostate tumors with extensive invasive adenocarcinoma, and were euthanized for bladder obstruction, with a median survival time of about 97 days post-tamoxifen treatment (or about 125 days of age) (Fig 5D and 5E). The final mouse was euthanized at 24 weeks after starting treatment and found to have HGPIN, but no invasive cancer. Examination of individual lobes suggested that the ventral prostate was most severely affected, with the anterior having the mildest phenotype (S6D Fig). We also tested different dosing schedules for the tamoxifen, which resulted in similar terminal phenotypes, but with much more variable lag times and lower penetrance (S6E and S6F Fig). As a comparison we treated mice with only conditional Pten alleles using the more robust treatment protocol and found that these mice had extensive HGPIN, but none had progressed to invasive cancer even by 40 weeks after treatment. Taken together, these data suggest that deletion of Pten and Tgfbr2 together specifically in luminal cells results in rapid progression to invasive cancer that is not seen with deletion of Pten alone.

### De-differentiation of luminal Pten;Tgfbr2 null cells

**Fig 6:**
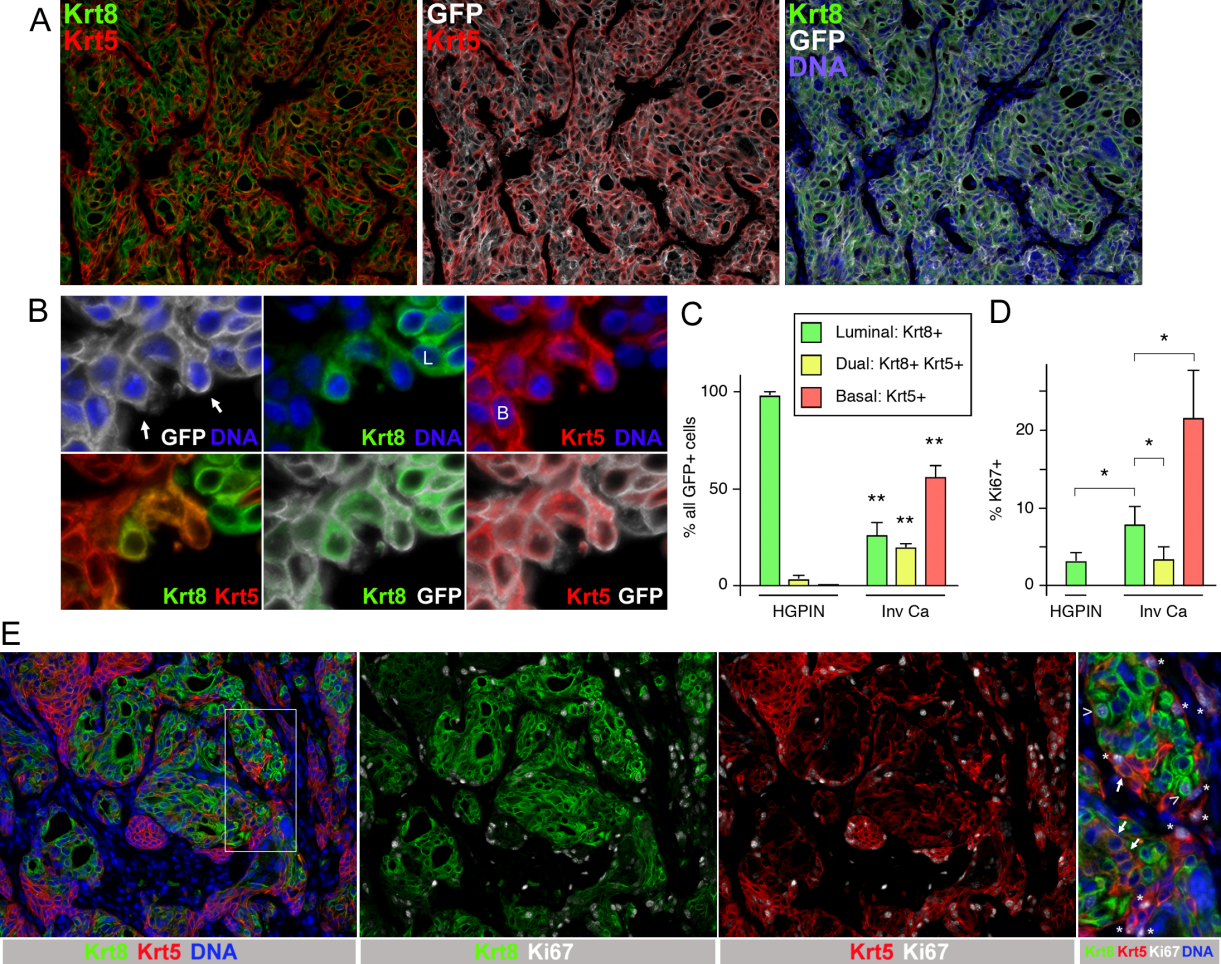
Increased numbers of dual positive cells in invasive cancer. A) Invasive cancer from a mouse at 12 weeks post-tamoxifen was analyzed by IF for Krt5, Krt8, GFP and DNA. Note the large amounts of basal cells (Krt5+) and dual positive cells (Krt8+/Krt5+) despite the initial luminal specific deletion. B) Higher resolution images of cells at the edge of an invasive region showing co-localization of Krt5 and Krt8 within individual cells (B: basal, L: luminal, arrows dual positive). C) Quantification of the proportions of luminal, basal and dual positive cells in regions of HGPIN and invasive cancer from Krt8-Cre Pten;Tgfbr2 mice euthanized for tumor burden. Only GFP positive cells were analyzed (three mice each, > 500 cells for HGPIN, 79–144 each for invasive cancer). D) Quantification of the proportion of proliferating (Ki67 positive) basal, dual positive and luminal cells in HGPIN and invasive cancer (four mice, 296–2250 cells each). E) A representative region of invasive cancer is shown, stained for Krt5, Krt8 and Ki67 (and for DNA). The region boxed in the left hand panel is shown at higher magnification, with Ki67 positive luminal cells (arrow heads) and basal cells (asterisks) indicated. Arrows show dual positive cells which in this region are negative for Ki67.

We next examined the cell types present in HGPIN and invasive cancer from the Krt8-Cre driven Pten;Tgfbr2 tumors. As shown in Fig 5C, prostate ducts with HGPIN from mice at 4–8 weeks after tamoxifen induction were exclusively Krt8 positive, with rare basal (Krt5 positive) cells that lacked GFP signal at the edges. However, when we examined invasive cancer from mice that had been euthanized for tumor burden, there was a considerable overlap between Krt5 and GFP signals (Fig 6A). More surprisingly, much of the Krt5 positive invasive cancer was also positive for Krt8, suggesting co-expression of both basal and luminal keratins. Closer examination of more isolated cells, where apparently overlapping keratin signals were unable to come from adjacent cells showed that both Krt5 and Krt8 were indeed expressed within the same cell, and that both signals were surrounded by the membrane targeted GFP recombination marker (Fig 6B). In advanced invasive cancer (all of which was GFP positive) we observed three distinct classes of keratin staining: Cells with high Krt8 and very low or absent Krt5 signal, as would be predicted from the Krt8-Cre driver, cells with high to intermediate levels of both Krt5 and Krt8, and those that had high Krt5 with little or no Krt8. Quantification of the number of basal (Krt5 high;Krt8 low/absent), luminal (Krt5 low/absent;Krt8 high) and dual positive cells from multiple tumors showed a significant increase in basal and dual positive cells in the invasive cancer compared to HGPIN, with a concomitant decrease in luminal cells (Fig 6C). Examination of invasive cancers from the Krt8-Cre model did not reveal any overt squamous differentiation, as seen in *Apc;Tgfbr2* null mouse prostate tumors, for example (S7A–S7C Fig) [19]. Gene expression analysis of PbCre Pten;Tgfbr2 tumors indicated extremely low expression of neuroendocrine markers, including synaptophysin. Similarly, analysis of synaptophysin in Krt8-Cre invasive tumors suggests that neuroendocrine differentiation in this model is extremely rare (S7D Fig). Thus it appears that luminal differentiation to dual positive and basal cells is the main effect of Tgfbr2 deletion in this model. We next examined proliferation by staining for Ki67 together with Krt5 and Krt8. As in the PbCre4 model, the majority of proliferating cells were basal rather than luminal in invasive cancer (Fig 6D and 6E). Proliferation in luminal cells increased from HGPIN to invasive cancer, with basal cell proliferation being even higher (Fig 6D). However, the proliferation rate of dual positive cells was not increased relative to luminal cells and was significantly lower than in basal cells, suggesting that the increase in their numbers is not due to rapid proliferation of rare dual positive cells present in HGPIN or early invasive tumors.

### Increased dual positive cells in early metastases

**Fig 7:**
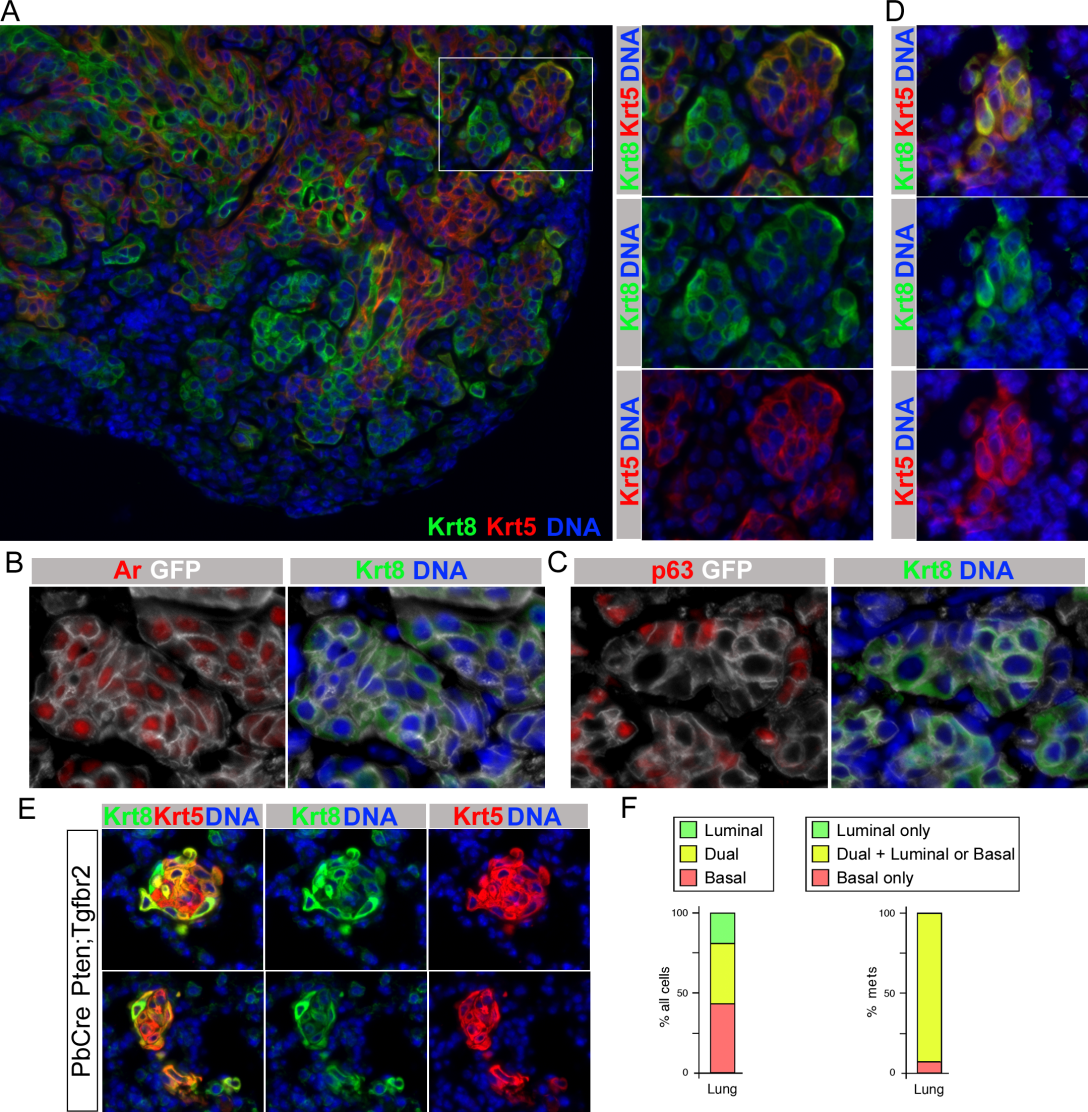
Analysis of cell types in lung metastases. A) A large lung metastasis from a Krt8-CreERT2 Pten;Tgfbr2 mouse stained for Krt5 and Krt8 is shown. The boxed region is magnified to the right, showing a mix of basal, luminal and dual positive cells, as in primary tumors. B) Lung metastasis staining for Krt8, Ar and GFP indicating prostate origin of the tumor. C) Krt8, p63, GFP staining indicating the presence of both basal and luminal cells in lung metastases. D) Krt5 and Krt8 staining of a lung micro-metastasis form a Krt8-CreERT2 Pten;Tgfbr2 mouse showing high proportion of dual positive cells. E) Two examples of lung micro-metastases from the PbCre4 Pten;Tgfbr2 model stained for Krt5, Krt8 and DNA. F) Quantification of the proportion of luminal, basal and dual positive cells in 42 lung micro-metastases (241 cells total) from PbCre4 Pten;Tgfbr2 mice. Left shows the percentage of individual cells of each type across all metastases. The right hand panel shows the proportion of metastases that were scored as either basal only, luminal only, or contained at least some dual positive cells, either exclusively or together with basal and or luminal cells.

In the lungs of six of seven Krt8-CreERT2 Pten;Tgfbr2 mice examined we identified metastases, which were positive for GFP. In larger metastases, we observed a mix of basal, luminal and dual positive cells that was similar to that seen in the primary tumors, as evidenced by staining for Krt5 and Krt8 (Fig 7A). Since the Krt8-CreERT2 driver is not specific to the prostate we also examined potential lung metastases for AR and Trp63. As shown in Fig 7B, GFP positive cells were positive for AR, with higher AR expression generally seen in Krt8+ GFP+ cells. The majority of GFP+ cells clearly expressed AR, with higher levels in Krt8+ cells, whereas no AR expression was detected in adjacent lung tissue (S8 Fig). A proportion of GFP positive cells within the lung were also Trp63 positive, and this signal was primarily distinct from Krt8+ cells consistent with these being basal cells (Fig 7C). This marker analysis indicates a similar expression pattern in lung tumors to that seen in the primary invasive prostate cancer. Thus metastasis from prostate is the most likely explanation for these GFP+ AR+ tumors in lung, although we cannot definitively rule out that some lung tumors are not derived from prostate metastases. While examining the cell types present in GFP positive lesions in the lungs of these mice, we noticed that most cells in lung micro-metastases were strongly positive for both Krt5 and Krt8, consistent with these smaller metastases being primarily composed of dual positive cells (Fig 7D). We previously reported frequent micro-metastases to the lungs of mice with prostate specific Pten and Tgfbr2 deletion using the PbCre4 driver [20]. In the primary tumors of these animals dual positive cells were very rare compared to the relatively high frequency seen in the Krt8-Cre model (see Fig 4B). Given the different balance of cell types in these two models, we examined the cell types present in lung micro-metastases from the PbCre4 tumors. As shown in Fig 7E, lung metastases frequently contained a large proportion of cells with high levels of both basal and luminal keratins. When we quantified this across 42 independent lung micro-metastases, we found that almost 40% of the cells examined were dual positive, with a similar proportion being basal, and only 20% luminal (Fig 7F). More than 90% of the metastases examined contained dual positive cells, with the remaining three metastases composed entirely of basal cells (Fig 7F). However, as we only examined a single section for each, it remains possible that other cell types were missed. Since the frequency of dual positive cells in the primary tumors in the PbCre4 model is very low, these data suggest that micro-metastases are either preferentially derived from this rare population, or that the cells that generate metastases have increased lineage plasticity once they seed the lung.

**Fig 8:**
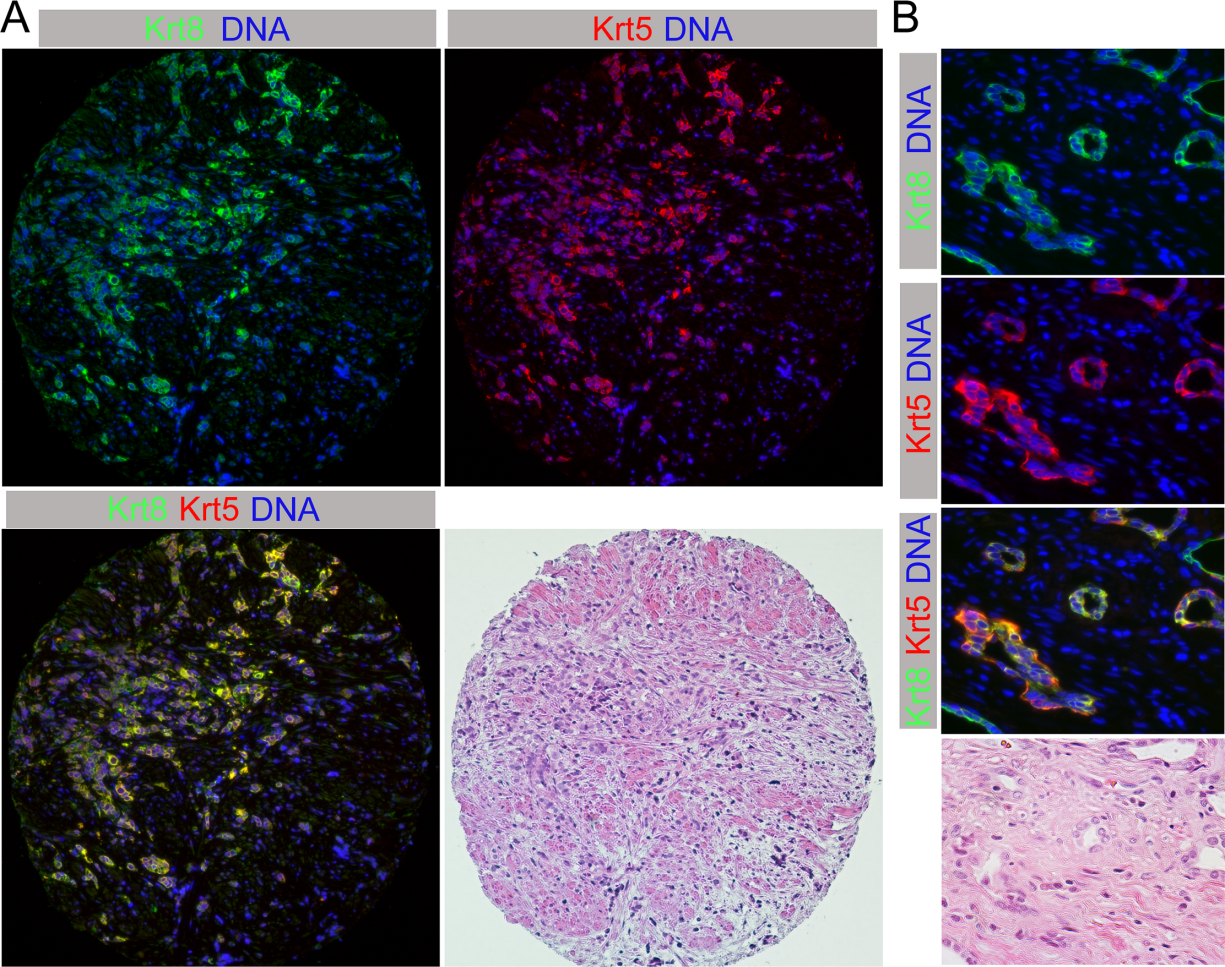
Dual positive cells in human CaP. A) A human prostate tumor, post androgen deprivation therapy is shown stained for Krt8 and Krt5, together with H&E staining of the same section. B) A second example of a human tumor with dual positive cells is shown at higher magnification.

Given that de-differentiated dual positive cells in these Pten;Tgfbr2 CaP models appear to drive invasion and metastasis, we examined whether cells with expression of both basal and luminal keratins were present in human CaP. We examined a panel of human CaP samples by IF for both Krt5 and Krt8. Of the 138 informative samples included on three tissue microarrays (21% Gleason 5–6, 68% Gleason 7–8, 11% Gleason 9–10), only one sample had tumor cells that were clearly dual positive (Fig 8A). Strikingly, this was the only prostate sample with marked atrophic changes consistent with prior androgen ablation therapy. Although it has not been common practice to undergo prostatectomy after androgen deprivation therapy (ADT), we obtained samples from ten additional patients who had had radical prostatectomy after some form of ADT. However, the types of drugs administered and their dosages and durations were quite variable. The range of beginning ADT to time of radical prostatectomy was between 2 weeks and 3 years (median: 19 months). Of these ten, two more contained dual positive cancer cells (Fig 8B). Although these dual positive cells were quite rare, the fact that we identified a small number of human cancers with tumor cells co-expressing both basal and luminal keratins is consistent with the idea that this is a potentially important cell type in human CaP. This notion is also supported by recent work showing increased lineage plasticity in *Pten* null tumors lacking both *Rb1* and *Trp53* [41].

## Discussion

Here we show that in the background of a Pten null mutation in mouse prostate, deletion of Tgfbr2 results in increased basal cell proliferation as tumors become invasive. Analysis of micrometastases from these mice suggests that cells expressing both luminal and basal keratins are more metastatic. Deletion of Pten and Tgfbr2 specifically from luminal cells also causes a rapid onset of invasive and metastatic cancer that is accompanied by a transition to a less differentiated cell type. We suggest a model in which TGFß signaling maintains a differentiated cell phenotype, and that loss of signaling may allow for de-differentiation to a more invasive and metastatic cell type.

Disruption of TGFß signaling either by Smad4 or Tgfbr2 deletion in the background of a prostate specific tumor initiating mutation results in rapid progression to invasive and metastatic cancer in mouse models [20, 24]. Transcriptome analysis suggested that this was accompanied by increased expression of basal cell specific genes, including Trp63 and basal keratins. This increase appears to be due to increased basal cell numbers in these tumors following Tgfbr2 deletion, and we show that a major effect of TGFß signaling in this context is to limit proliferation of basal cells. Given the results from the luminal-specific deletion model, some de-differentiation of luminal to basal cells may also contribute, although this is likely a minor component in the PbCre4 driven tumors. Previous work has also suggested a role for TGFß signaling, in conjunction with the Notch pathway, in limiting basal cell proliferation [51]. Analysis of gene expression data suggests that in comparison to the Pten null tumors, disruption of TGFß signaling primarily affects expression of gene sets associated with highly proliferative, aggressive cancers. Although gene sets associated with EMT or invasion are enriched in the tumors compared to wild type prostate, it appears that such invasive gene expression signatures are already enriched by deletion of Pten alone. Although deletion of Pten from mouse prostate results in invasive cancer, it does so relatively slowly. Regions of micro-invasion may be detectable early, but large locally invasive cancers are not generally detected in the Pten model until 30–50 weeks of age, and metastases are extremely rare [19, 20]. Thus, the combination of a local micro-invasive phenotype initiated by Pten loss, together with increased proliferation due to loss of Tgfbr2 drives rapid progression to invasive cancer. However, this model is complicated by the loss of Pten and Tgfbr2 from both basal and luminal cells, together with the dramatic effect of the Tgfbr2 deletion specifically on basal cell proliferation.

Human CaP primarily consists of malignant luminal epithelial cells, with other subtypes, including basaloid and neuroendocrine cancers being much less frequent [31]. Recent evidence has renewed interest in understanding the neuroendocrine phenotype, as it may be part of the mechanism by which tumors escape the effects of anti-androgen therapy [40, 41]. Despite the predominance of the luminal phenotype in human CaP, there is considerable evidence that both basal and luminal cells can be the cell of origin for cancer [32–36]. To more accurately model tumors in which a single cell type is the target for oncogenic transformation, we used a luminal-specific Cre driver [50]. Deletion of Pten using a tamoxifen inducible Krt8-Cre transgene that is active specifically in luminal cells resulted in HGPIN, consistent with previous reports [32]. When combined with a conditional Tgfbr2 allele we observed a rapid progression to invasive and metastatic cancer, which was not seen in Pten single mutants, even at advanced age. Given that the primary effects of Tgfbr2 deletion using the PbCre4 model appeared to be in basal cells, the rapidity with which these tumors progressed was somewhat unexpected. The PbCre4 transgene begins to drive recombination at around 4 weeks of age [52], and extensive phenotypes were clearly detectable by 8 weeks. In our previous work, Pten;Tgfbr2 double mutants had a median survival of around 12–13 weeks [20]. Using the Krt8-CreERT2 driver here, and initiating tamoxifen treatment at 4 weeks after birth, median Pten;Tgfbr2 double mutant survival was 17–18 weeks of age, or 13–14 weeks after initiating tamoxifen treatment. This represents an approximately 50% increase in lifespan that may be due to the fact that only a fraction of luminal cells undergo recombination, initially resulting in smaller tumors. Although, it is possible that differences in strain background might also contribute to this difference in survival. However, this clearly shows that disrupting TGFß signaling specifically in luminal cells can drive progression to invasive cancer. Other tamoxifen dosing regimens resulted in similar phenotypes with reduced penetrance and longer lag times, although we did not attempt to induce recombination in very old animals.

The Krt8-CreERT2 driver used here demonstrates recombination that is highly specific to luminal cells in the prostate [29]. We searched extensively for basal cells in which recombination of the lineage tracing reporter could be detected early after tamoxifen treatment and did not find any evidence for recombination in Krt5 positive basal cells. Despite this, it is clearly possible that rare basal or uncommitted prostate epithelial cells may undergo recombination driven by this transgene. However, given our inability to identify such cells and the rapid onset of the phenotypes observed here we do not think that such inappropriate recombination contributes significantly to the phenotypes observed. Since basal cell proliferation is so rapid in the absence of Tgfbr2, if rare basal cells underwent recombination we would expect to identify small focal regions of basal cells within the tumors, without the relatively high proportion of cells expressing both basal and luminal keratins. It should be noted that these dual positive cells have a relatively low proliferation rate, certainly much less than that of basal cells and even lower than Pten;Tgfbr2 null luminal cells. If these cells arise from rare recombination events in uncommitted progenitors, or rare dual positive cells, it seems unlikely that they would become such a large proportion of the tumor given that their proliferation rate is lower than that of other cell types. Thus, the generation of dual positive cells in these tumors most likely occurs by differentiation of either luminal or basal cells to a more intermediate cell type. Since these dual positive cells have a lower proliferative rate than either basal or luminal cells, it is tempting to speculate that this may be linked to their apparently higher invasive ability. The fact that we see both dual positive and basal cells at high frequency in invasive cancers, while HGPIN is exclusively luminal suggests that both dual positive and basal cells arise from luminal cells lacking Pten and Tgfbr2. The relatively low proliferation rate of dual positive cells suggests that de-differentiation to this cell type must be a relatively frequent event in this tumor model. This suggests that TGFß signaling in the prostate acts to maintain the differentiated phenotype of luminal cells, and that loss of TGFß signaling in CaP may contribute to lineage plasticity. Interestingly, recent work has shown that TGFß signaling may also limit de-differentiation in intestinal tumors [53], suggesting that this may be a more common role of TGFß signaling in cancer progression.

Recent work has suggested that lineage plasticity may contribute to CaP recurrence following anti-androgen therapy [40, 41]. One possibility is that relatively rare multipotent cells may be able to survive androgen ablation and allow subsequent relapse even after the majority of the tumor, which has a primarily luminal phenotype, has regressed. Thus, therapeutic intervention might select for pre-existing cells that are more able to withstand the treatment and retain the ability to differentiate to luminal or other cell types. In addition, there has been interest in so called castration resistant Nkx3-1 expressing cells (CARNs). These cells may represent a similar population that is derived from luminal cells, and can be effectively selected for by androgen deprivation and restoration in mouse models [33, 34]. If this is a mechanism by which CaP can recur, then it would be of significant interest to understand what molecular pathways promote the existence or maintenance of such de-differentiated cells within the tumor. Our data suggest that TGFß signaling acts to maintain the differentiated state of luminal tumor cells and that loss of this signaling pathway allows de-differentiation to an intermediate cell type, which can then further differentiate to a basal cell phenotype. While the final differentiation to basal cells as seen in our model may not be the usual situation in human CaP, the generation of de-differentiated intermediate cells might provide insight into how these cells arise in human CaP. In this context, it is worth noting that in the extensively basal Pten;Tgfbr2 null invasive tumors from the PbCre4 model, there is enrichment for gene expression signatures that are indicative of aggressive luminal cancers.

The rapid basal cell proliferation in both the PbCre4 and Krt8-CreERT2 models places some limitation on them as far as being representative of human CaP, since basaloid carcinoma of the prostate is quite rare [31]. As with many mouse models of human cancer, one potential confounding issue is the complete inactivation of a particular gene (Tgfbr2 in this case). In contrast, in the human disease changes in expression or activity may be more graded, resulting in reduced activity rather than complete loss of function. Although recurrent inactivating mutations or deletions of the major components of the TGFß signaling pathways are not found in human CaP, there is evidence for reduced expression of both TGFBR1 and TGFBR2 in more advanced CaP [12–14], and reduced SMAD4 and TGFBR2 expression due to promoter methylation [9, 11]. Low SMAD4 expression was found to be part of a four gene signature that was prognostic for CaP recurrence and metastasis [24]. Our analysis of SMAD4 and active phosphorylated SMAD2 in human CaP also supports the idea that pathway activity may be dampened rather than completely abrogated in human CaP. While the mouse models used here are not ideal for addressing all questions regarding advanced human CaP, they may uncover important features of earlier stages of disease progression and metastasis. In the Krt8-CreERT2 model luminal tumor cells lacking Pten and Tgfbr2 de-differentiate to dual positive cells, a process that may be of interest for understanding human CaP progression. While loss of TGFß signaling may not be the only way to drive this de-differentiation, understanding this transition could contribute to the development of better therapeutic approaches. Recent work with mouse models and xenografts has suggested that androgen ablation selects for a more de-differentiated cell type with increased lineage plasticity in CaP [40, 41, 54]. This lineage plasticity may underlie the ability of human CaP to escape anti-androgen or anti-AR therapies, ultimately resulting in therapy failure. Analysis of human CaP phenotypes from before and after the introduction of newer anti-AR therapies, such as enzalutamide and abiratirone, suggests an increase in the frequency of AR negative tumors after anti-AR therapy [55]. The majority of these AR negative recurrent tumors lacked neuroendocrine features, and this transition could be mimicked by shutting off AR expression in the human LNCaP cell line. Interestingly, AR negative LNCaP cells gained expression of some basal markers, while retaining expression luminal-specific genes [55]. These studies clearly support the notion that current therapies for human CaP may fail in part due to the presence of de-differentiated tumor cells that do not rely on androgens for survival. These cells may initially be rare, or only exist in readily detectable numbers transiently following therapy. Once the recurrent disease has progressed to a more advanced stage such intermediate cells will likely have differentiated, either to a neuroendocrine phenotype [40, 41, 54], or to androgen-independent non-neuroendocrine tumors [55]. While the Krt8-CreERT2 model used here results in large numbers of de-differentiated dual positive cells, one limitation is that these tumors are overtaken by basal cells, possibly derived from the dual positive population. We do not know if interfering with androgen signaling affects the generation of dual positive cells in this model, but this may be of interest given the possibility that anti-androgen therapy selects for de-differentiated cells in human CaP.

A major question raised by our work is how frequently dual positive cells are found in human CaP samples, and what role they might play in tumor progression. Our analysis of human tumors is consistent with the idea that if dual positive cells are present, they are extremely rare in tumors prior to ADT. Indeed, we only identified dual positive cells in tumors that had undergone ADT. The frequency of post-ADT tumors with dual positive cells was more than 25% (3 of 11 samples), despite the heterogenous modes of ADT, and the variable time after treatment that the samples were isolated. Although this is based on a very small sample size, the identification of any tumors with dual positive cells, together with the evidence for increased lineage plasticity in post-ADT tumors, is quite intriguing. This population may represent an initially very rare cell type that is selected for by ADT, but is present only transiently prior to transition to a more differentiated cell type in the recurrent tumor. Clearly, it will be important to examine additional post-ADT tumors for the presence of dual positive cells to better determine how frequently they are present, and to further interrogate the molecular features of such cells. With a larger cohort of tumors bearing dual positive cells, it might be possible to identify pathways that promote this cell type in human CaP. Together, our data and that from other mouse CaP models, suggest that luminal cell differentiation to an intermediate cell type may be important for driving invasion, metastasis and disease recurrence. Intermediate cells may be selected for by androgen depletion, or may be promoted by other mechanisms, such as loss of Trp53 and Rb1, or by reduced activity of the TGFß pathway [40, 41, 54]. In human tumors, reduced Trp53, Rb1 or TGFß signaling might facilitate the selective effects of ADT on cells with increased linage plasticity.

Our analysis of metastases in the Krt8-CreERT2 model suggests that larger lung tumors resemble the primary prostate tumors, expressing the AR at varying levels and Trp63 in a proportion of the cells. This is clearly consistent with the notion that these are indeed prostate-derived metastases, although we cannot definitively rule out other origins. In addition, these lung metastases have a mixture of basal, luminal and dual positive cells as seen in the primary tumors. However, our examination of smaller metastases in the PbCre4 and Krt8-CreERT2 models suggests that a larger proportion of the cells within these micro-metastases are dual positive. This is particularly striking in the PbCre4 model, in which lung metastases must be from prostate. In this model a very low proportion of the primary tumor is dual positive. In contrast, more than one third of cells in the micro-metastases are dual positive and almost all micro-metastases identified contained dual positive cells. This suggests that these cells may be more metastatic, or that cells that are competent for metastasis are also able to de-differentiate, further emphasizing the potential importance of such highly plastic cells to disease progression. In this context, it is interesting to note that the widespread appearance of dual positive cells in the Krt8-CreERT2 model roughly coincides with the onset of extensive locally invasive cancer. Even in a single tumor, ducts with HGPIN are purely luminal whereas adjacent regions of invasive cancer frequently contain a mix of luminal, basal and dual positive cells. One question this raises is why, if TGFß limits plasticity, the cells within a relatively intact duct do not undergo de-differentiation. One possibility is that the initial invasion out of the duct exposes the cells to other signals that in the absence of a luminal cell TGFß response are able to drive de-differentiation.

In summary, we show that loss of TGFß signaling specifically from Pten null luminal prostate cells allows for rapid progression to an invasive and metastatic phenotype that is accompanied by de-differentiation. We suggest that this may be analogous to the proposed lineage plasticity seen in human CaP following androgen ablation or in other mouse models in which Pten and Rb1 have been deleted. Our analysis of human samples suggests that dual positive cells can be found in human CaP, potentially only after ADT. Importantly, by combining luminal specific deletion, lineage tracing and extensive analysis of basal and luminal markers, we provide evidence for a transition from luminal tumor cells to a de-differentiated invasive cell type that may be important for metastatic spread.

## Materials and methods

### Ethics statement

All animal procedures were approved by the Animal Care and Use Committee of the University of Virginia, which is fully accredited by the AAALAC. The use of archival human specimens was approved by the University of Virginia IRB (HSR# 13310; Identification of biomarkers in diseased human specimens). Under this protocol no patient consent was required, as samples were archival leftovers from diagnostic samples with patient identifiers not released to the principal investigator.

### Mice

The *loxP* flanked *Pten*, *Apc* and *Tgfbr2* alleles and the *PbCre4* allele have been described previously [52, 56–58]. *Tgfbr2* and *Apc* mice, and the *PbCre4* transgenics were obtained from the NCI. The Krt8-CreERT2 line was from Jax (#017947 [50]). Conditional alleles with loxP flanked exons, which when recombined result in null alleles, and are referred to here as ‘r’ for recombined (null), or ‘f’ for the conditional loxP flanked (equivalent to wild type). Mice with a lineage tracing reporter (mTmG; #007576) were obtained from Jax [49]. All mouse lines were maintained on a mixed C57BL/6J x FVB strain background, as previously described [19, 20]. Tamoxifen treatment was by gavage, using 200mg/kg tamoxifen in corn oil daily for five days, or two sets of five days separated by a two day break. Genomic DNA for PCR genotype analysis was purified from ear punch, at post-natal day 21 (P21), by HotShot [59], and genotypes were determined by PCR. TRAMP tumor samples were as in [60].

### RNA isolation and qRT-PCR

RNA was isolated and purified using Absolutely RNA kit (Agilent) and quality checked by Bioanalyzer. For RNA isolation ventral lobes were used in all cases where the lobes were still distinguishable. For larger invasive tumors, a portion of the tumor was taken, although in most cases the lobes were not distinguishable. cDNA was generated using Superscript III (Invitrogen), and analyzed in triplicate by real time PCR using a BioRad MyIQ cycler and Sensimix Plus SYBRgreen plus FITC mix (Bioline), with intron spanning primer pairs, selected using Primer3 (http://frodo.wi.mit.edu/). Expression was normalized to Rpl4 and Cyclophilin using the delta Ct method.

### RNA-sequencing and analysis

Poly-A RNA-seq libraries generated with Illumina barcodes were sequenced (Illumina HiSeq at HudsonAlpha) to a target depth of ~ 25M paired end 50bp reads per sample as previously described [61]. Raw FASTQ sequencing reads were chastity filtered and reads were assessed for quality using FastQC. Reads were splice-aware aligned to the reference genome using STAR [62], and reads overlapping gene regions were counted using featureCounts [63]. The DESeq2 Bioconductor package [64] in the R statistical computing environment (http://www.R-project.org/) was used for normalizing count data, performing exploratory data analysis, estimating dispersion, and fitting a negative binomial model for each gene comparing the expression from tumors to wild type samples. Benjamini-Hochberg FDR was used to correct p-values for multiple testing. A cut-off of +/- 1.0 log2 and an adjusted P-value of < 0.0001 was considered significant. GO analysis was performed using either DAVID (https://david.ncifcrf.gov/) [65, 66] or ENRICHR (http://amp.pharm.mssm.edu/Enrichr/) [67, 68], heat maps were generated using ClustVis (http://biit.cs.ut.ee/clustvis/) [69] and gene set enrichment was performed using GSEA software from the Broad Institute [70, 71]. RNA-seq data is deposited at GEO, under the accession number: GSE108017.

### Histology and IF analysis

Tissues were fixed in zinc-formalin, paraffin-embedded and sectioned at 5 microns, and were stained with Hematoxylin and Eosin (H&E), or prepared for immunostaining as previously described [61, 72]. Images were captured with 10, 20 or 40x objectives, using a Nikon Eclipse NI-U with a DS-QI1 or DS-Ri1 camera and NIS Elements software, and adjusted in Adobe Photoshop. Antibodies were as follows: Rabbit and chicken anti-Krt5, mouse anti-Krt8 (Covance), rabbit anti-Krt8 (Abcam), rabbit anti-Ki-67 (Abcam), rabbit anti-AR (AR-441; Abcam), mouse anti-p63 (Biocare medical), rabbit anti-Syp (Thermo Fisher Scientific), rabbit anti-Krt10 (Covance), rabbit anti-Tgfbr2 (Novus), mouse anti-pAkt (Cell Signaling), rabbit anti-pSmad2 (Millipore), rabbit anti-Smad4 (Millipore) and chicken anti-GFP (Abcam). Alexafluor 488, 546 and 647 secondary antibodies were from Invitrogen. DNA was stained with Hoechst 33342. Confocal images were captured using a Zeiss LSM710 Multiphoton Confocal microscope and adjusted in Adobe Photoshop. Two tissue microarrays (TMA) contained four 0.6mm tissue cores from each of 93 cancers from patients who had radical prostatectomy. A third TMA contained four 0.6mm cores from each of 45 cancers from TURP samples. TMAs were examined by IHC, as in [20], or by IF for Krt5 and Krt8. Following IF and imaging, the same slide was stained with H&E.

## Supporting information

S1 Fig Analysis of pAKT and the TGFß pathway in human CaP. Examples of IHC for pAKT (A), SMAD4 (B) and pSMAD2 (C) are shown. For each, representative high and low staining samples are shown. D) Summary of staining patterns for pAKT, SMAD4 and pSMAD2 with Gleason score for 38 human tumor samples. E) The data for IHC score is shown separated first by pAKT (high/low), then SMAD4 (high/low), and finally pSMAD2 (high/low). A total of 9 of the 38 samples (blue numbers) are high for both SMAD4 and pSMAD2.
(TIF)

S2 Fig GSEA of gene signatures for aggressive highly proliferative cancers. A) GSEA comparing P-22 to wild type, PT-11 to wild type and PT-11 to P-22 tumors, showing enrichment for the CIN70 gene-set, Foxm1 targets and an ESC like signature from aggressive tumors. B) Overlap between the three gene sets analyzed in A.
(TIF)

S3 Fig Comparison of Krt5 staining in invasive Pten and Pten;Tgfbr2 null tumors. A representative image of an invasive Pten tumor (at 45 weeks of age) stained for Krt8 and Krt5 is shown, together with a Pten;Tgfbr2 (12 week invasive tumor) for comparison.
(TIF)

S4 Fig Pten;Tgfbr2 null tumors are enriched for gene expression signatures associated with aggressive luminal cancer. A) Heat-map showing relative expression for a 50 gene set that distinguishes luminal A, luminal B and basal like tumor subtypes in prostate cancer. Genes indicative of each subtype are color coded to the left–note the enrichment for high expression of Luminal B associated genes in the double null tumors. B) Heat-map showing comparison to a 37 gene signature that distinguishes two luminal subtypes (SEG1 and SEG2) and one basal like subtype (SEG3). Genes indicative of the more aggressive luminal SEG1 are enriched in Pten;Tgfbr2 null tumors. C) Comparison to the larger data sets for SEG1, 2, 3. The number of genes in each group is shown, as well as the number (and percentage of total) that increase or decrease significantly in the indicated comparisons between our RNA-seq data-sets.
(TIF)

S5 Fig Luminal specific recombination with Krt8-CreERT2. A) The upper panels show a series of confocal slices of prostate stained for GFP and Krt5 four weeks after tamoxifen treatment. The boxed region is shown at higher resolution to the lower left (overlaid image and individual channels), and the region boxed in this image is further magnified at the lower right. B) Staining for pAkt and GFP in a prostate 6 weeks after tamoxifen, showing overlap of pAkt (indicative of Pten loss) and GFP. C) Staining for Tgfbr2 and GFP in a prostate 6 weeks after tamoxifen, showing a lack of overlap of Tgfbr2 and GFP, consistent with deletion of Tgfbr2 and GFP activation in the same cells.
(TIF)

S6 Fig Analysis of recombination and phenotypes following tamoxifen treatment. A) 143 ducts (selected randomly without first visualizing GFP staining) in mice 4–6 weeks after tamoxifen were scored for the proportion of cells that were GFP positive. The mean + sd is shown above for all ducts and separately for those with PIN or without phenotype. Below is the distribution of GFP positive cells per duct (excluding ducts without any GPF cells). Plotted as median, 25^th^ and 75^th^ percentiles (box) and 5^th^ and 95^th^ percentiles (whiskers). B) The proportion of normal and PIN ducts with GFP positive cells is shown. C) The proportion of GFP negative and GFP positive ducts with PIN is shown. D) The proportion of mice with either HGPIN or invasive cancer as the worst phenotype in each lobe (anterior [AP], ventral [VP] or dorsolateral [DLP] prostate) is shown, from the Pten;Tgfbr2 mice analyzed for survival in Fig 5D. E) Survival analysis for Krt8-CreERT2 Pten;Tgfbr2 mice treated either with one round of five days tamoxifen at four weeks (4w, 1x) or two rounds at six weeks (6w, 2x) of age is shown. F) Proportion of mice with either HGPIN or invasive cancer as the worst phenotype (in any lobe) at euthanasia for each treatment regimen (four weeks with one or two rounds: 4x1, 4x2 and six weeks with two rounds: 6x2), and for Pten single mutants is shown.
(TIF)

S7 Fig Phenotype analysis in invasive tumors. A) A Krt8Cre invasive double null tumor was stained for Krt5, Krt8 and Krt10 to examine squamous differentiation. B) An Apc;Tgfbr2 null tumor was analyzed for comparison–note the Krt10 signal in this tumor, which has squamous differentiation. C) High power (40x) image of H&E staining of an invasive Krt8Cre Pten;Tgfbr2 null tumor. D) A Krt8Cre invasive double null tumor was stained for Krt8 and Synaptophysin (Syp) to examine neuroendocrine differentiation. The region shown is representative of the highest Syp signal seen in these tumors. E) A TRAMP tumor is shown stained for Krt8 and Syp as a control for the neuroendocrine phenotype.
(TIF)

S8 Fig Extensive Ar staining in lung tumors. A lung tumor from a Krt8Cre invasive double null mouse was stained for Ar, Krt8 and GFP. Note that the majority of GFP cells are positive for Ar, with higher Ar signal generally associated with high Krt8 signal. This is consistent with the staining pattern seen in primary prostate tumors, consistent with lung tumors being prostate metastases.
(TIF)

S1 Table Transcriptome analysis of mouse prostate tumors. A) Average gene expression levels (reads per million) for each group of tumors, levels normalized to the wild type (set = 1), and FDRs for all pairwise comparisons are shown. B) Gene lists for all comparisons to wild type, selected using a 5 reads per million minimum (in at least one group), a log2-fold change of +/-1.0 and a p-adjusted of < 0.0001. C) Summary tables showing GO analyses and comparisons to ENCODE and ChEA data, generated using ENRICHR.
(XLSX)

S2 Table Data for graphs in figures. The numerical data for graphs presented in Figures 2, 3, 4, 6 and 7 are shown.
(XLSX)
