# Supplementary figures and images for: TGFβ signaling limits lineage plasticity in prostate cancer
Source: PLoS Genet. 2018 May 21;14(5):e1007409. doi: 10.1371/journal.pgen.1007409 (PMC5983872; doi:10.1371/journal.pgen.1007409)

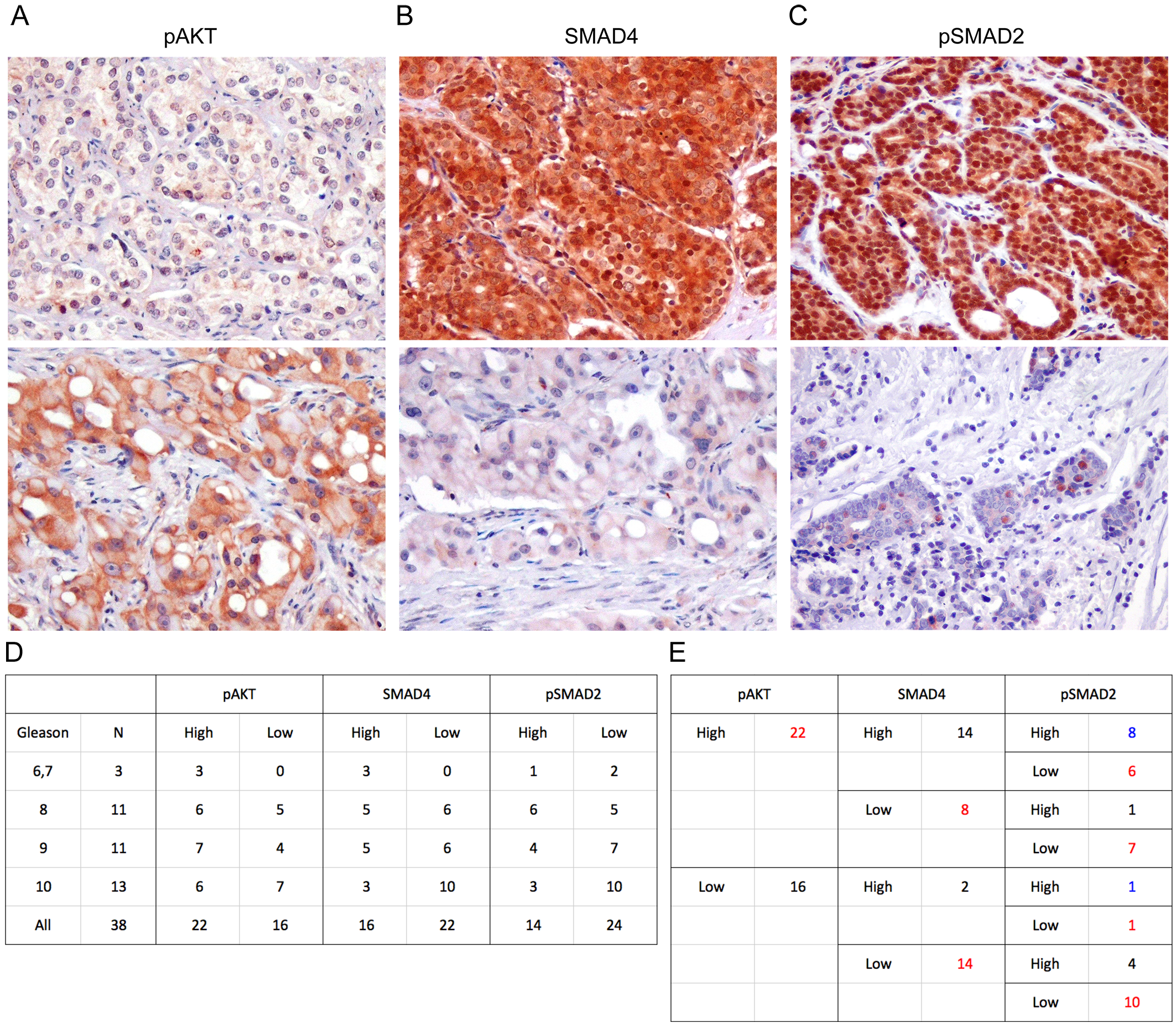

Supplement: S1 Fig — Examples of IHC for pAKT (A), SMAD4 (B) and pSMAD2 (C) are shown. For each, representative high and low staining samples are shown. D) Summary of staining patterns for pAKT, SMAD4 and pSMAD2 with Gleason score for 38 human tumor samples. E) The data for IHC score is shown separated first by pAKT (high/low), then SMAD4 (high/low), and finally pSMAD2 (high/low). A total of 9 of the 38 samples (blue numbers) are high for both SMAD4 and pSMAD2. (TIF) [file pgen.1007409.s001.tif]

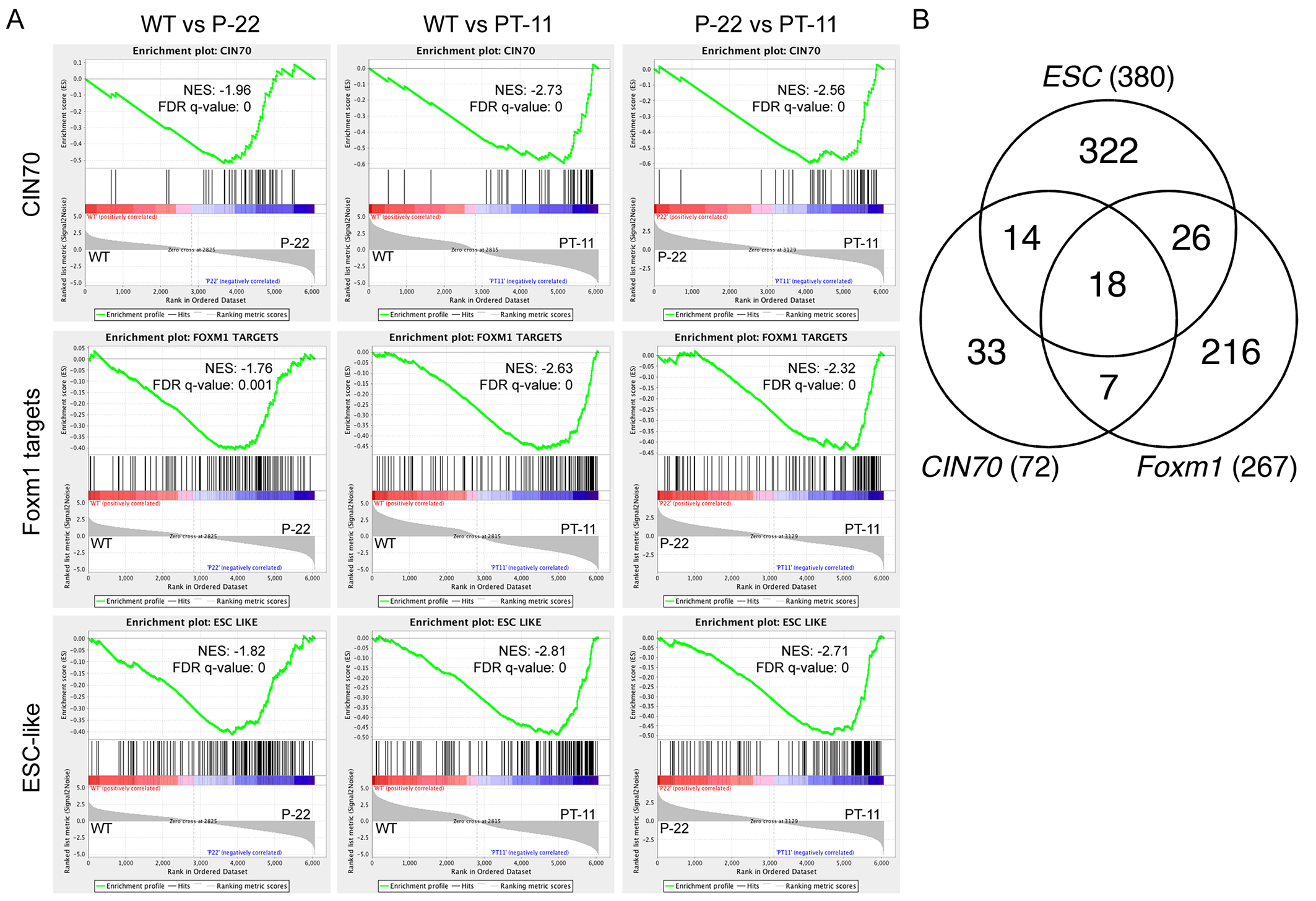

Supplement: S2 Fig — A) GSEA comparing P-22 to wild type, PT-11 to wild type and PT-11 to P-22 tumors, showing enrichment for the CIN70 gene-set, Foxm1 targets and an ESC like signature from aggressive tumors. B) Overlap between the three gene sets analyzed in A. (TIF) [file pgen.1007409.s002.tif]

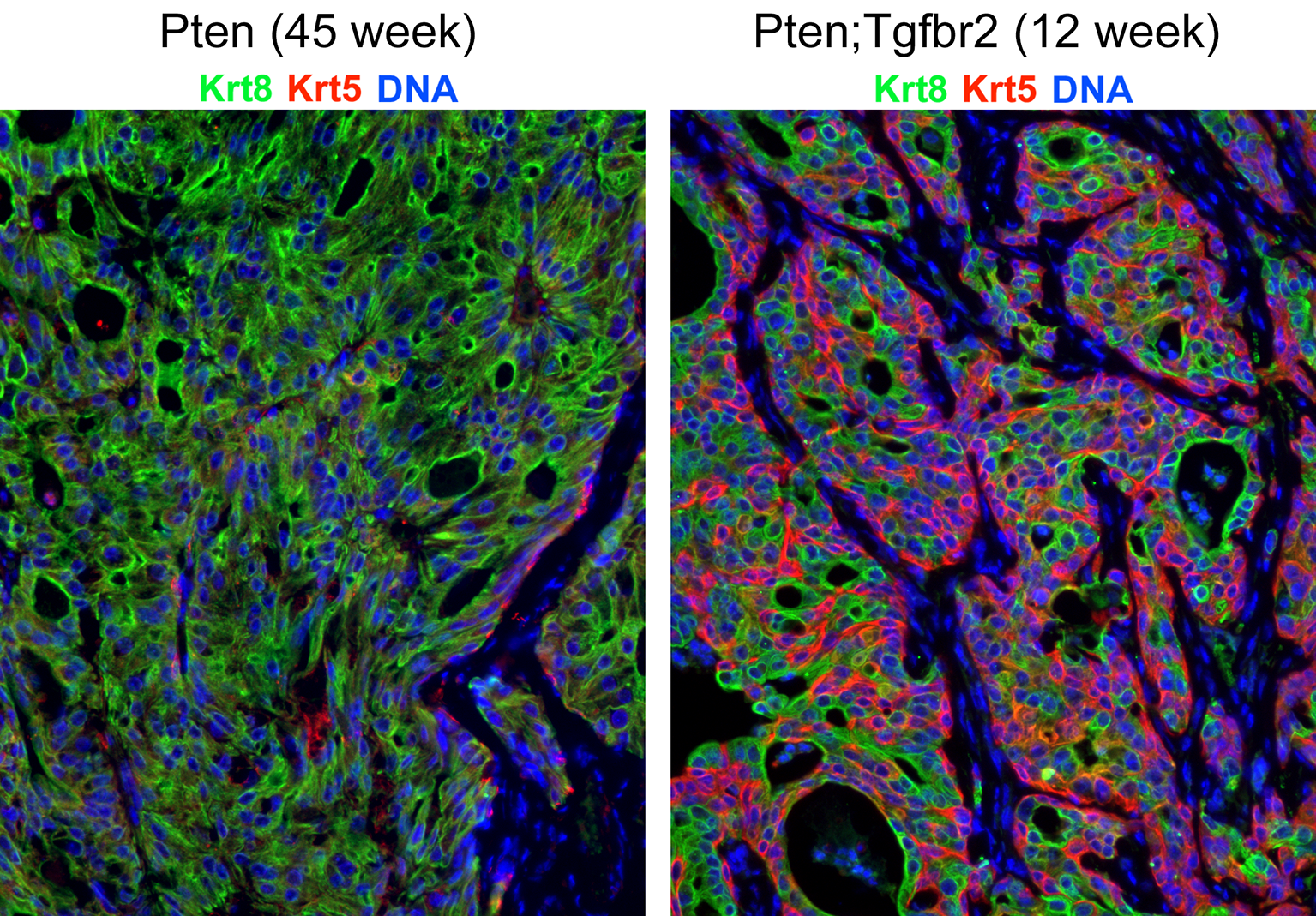

Supplement: S3 Fig — A representative image of an invasive Pten tumor (at 45 weeks of age) stained for Krt8 and Krt5 is shown, together with a Pten;Tgfbr2 (12 week invasive tumor) for comparison. (TIF) [file pgen.1007409.s003.tif]

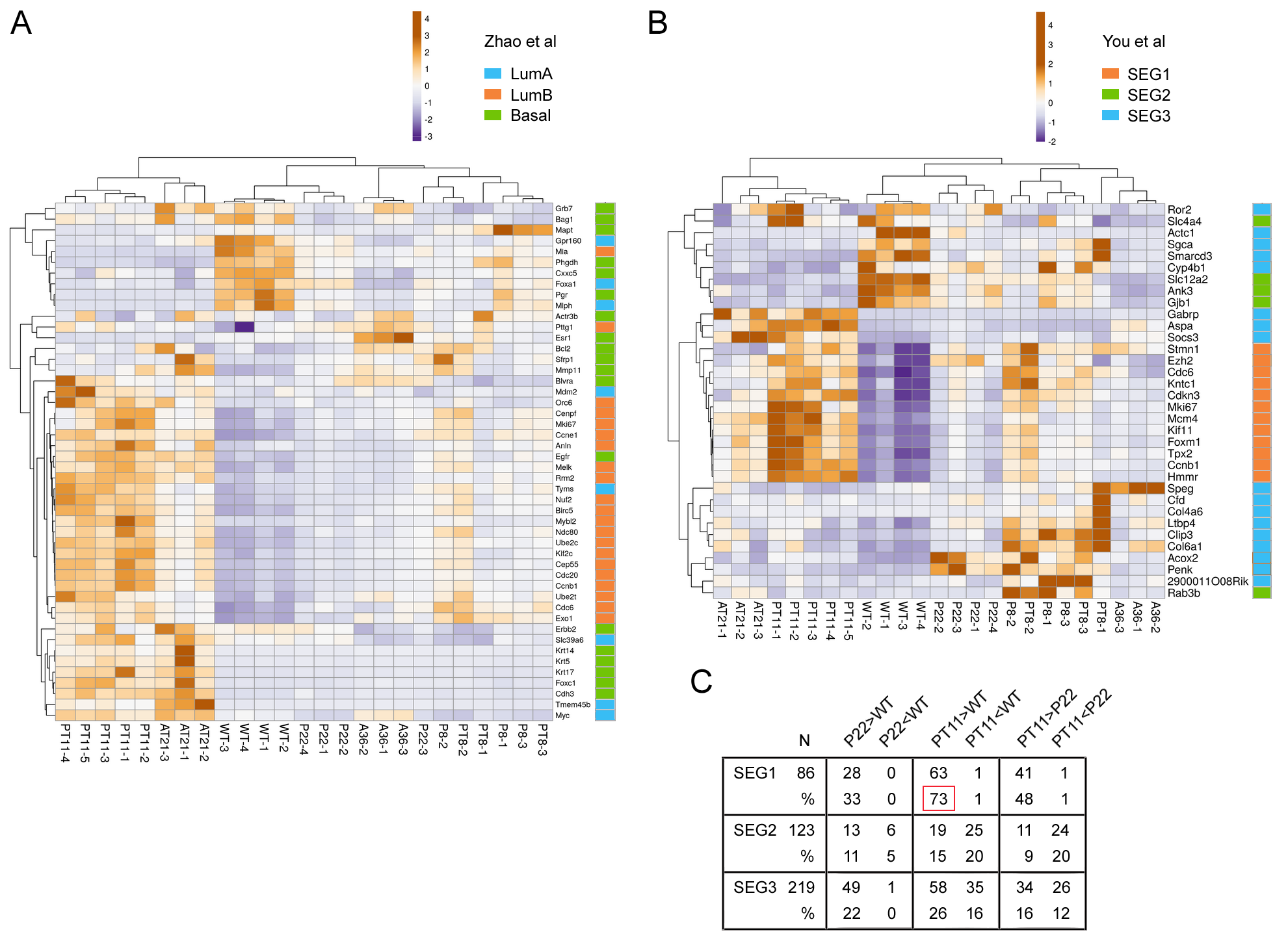

Supplement: S4 Fig — A) Heat-map showing relative expression for a 50 gene set that distinguishes luminal A, luminal B and basal like tumor subtypes in prostate cancer. Genes indicative of each subtype are color coded to the left–note the enrichment for high expression of Luminal B associated genes in the double null tumors. B) Heat-map showing comparison to a 37 gene signature that distinguishes two luminal subtypes (SEG1 and SEG2) and one basal like subtype (SEG3). Genes indicative of the more aggressive luminal SEG1 are enriched in Pten;Tgfbr2 null tumors. C) Comparison to the larger data sets for SEG1, 2, 3. The number of genes in each group is shown, as well as the number (and percentage of total) that increase or decrease significantly in the indicated comparisons between our RNA-seq data-sets. (TIF) [file pgen.1007409.s004.tif]

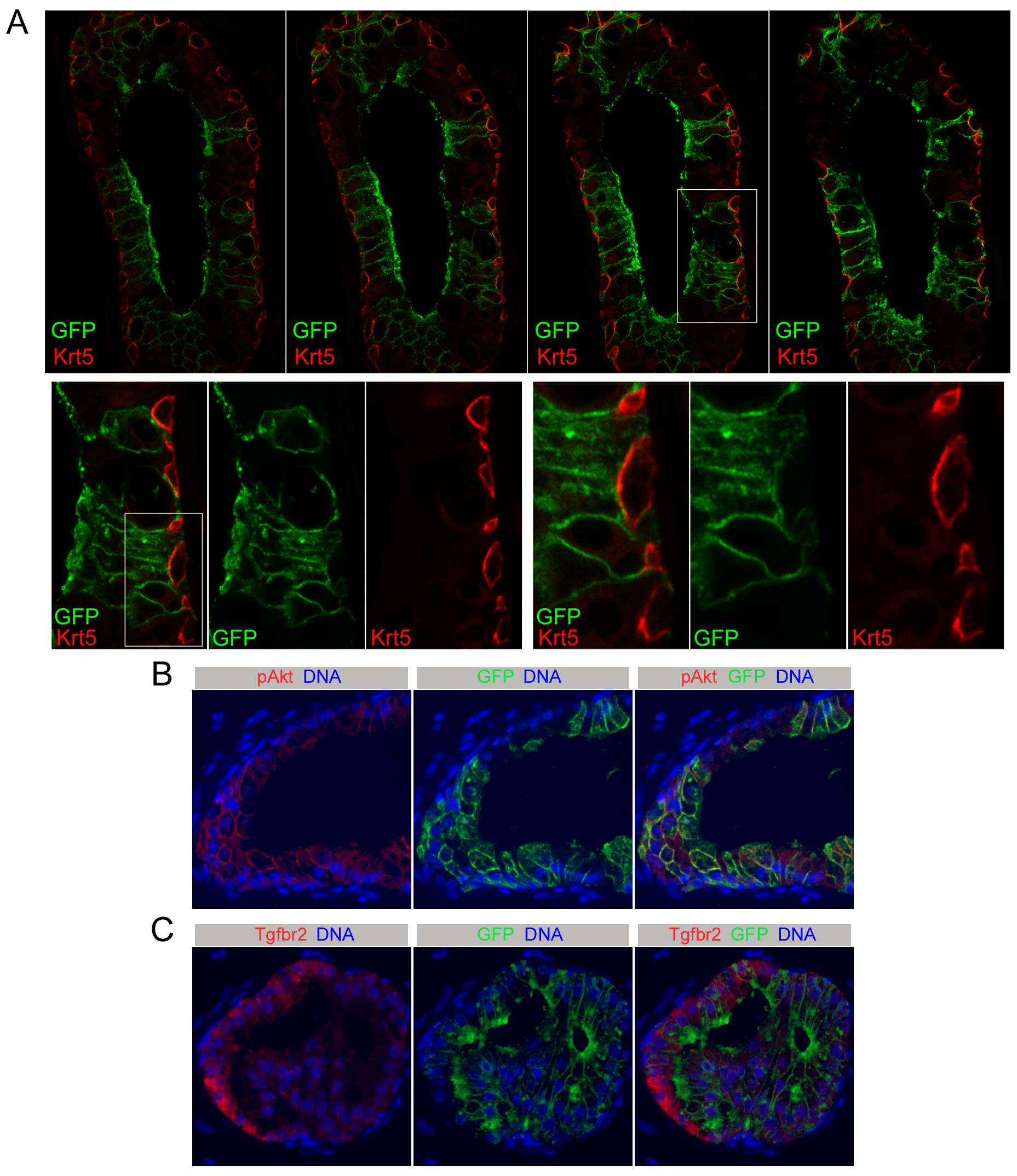

Supplement: S5 Fig — A) The upper panels show a series of confocal slices of prostate stained for GFP and Krt5 four weeks after tamoxifen treatment. The boxed region is shown at higher resolution to the lower left (overlaid image and individual channels), and the region boxed in this image is further magnified at the lower right. B) Staining for pAkt and GFP in a prostate 6 weeks after tamoxifen, showing overlap of pAkt (indicative of Pten loss) and GFP. C) Staining for Tgfbr2 and GFP in a prostate 6 weeks after tamoxifen, showing a lack of overlap of Tgfbr2 and GFP, consistent with deletion of Tgfbr2 and GFP activation in the same cells. (TIF) [file pgen.1007409.s005.tif]

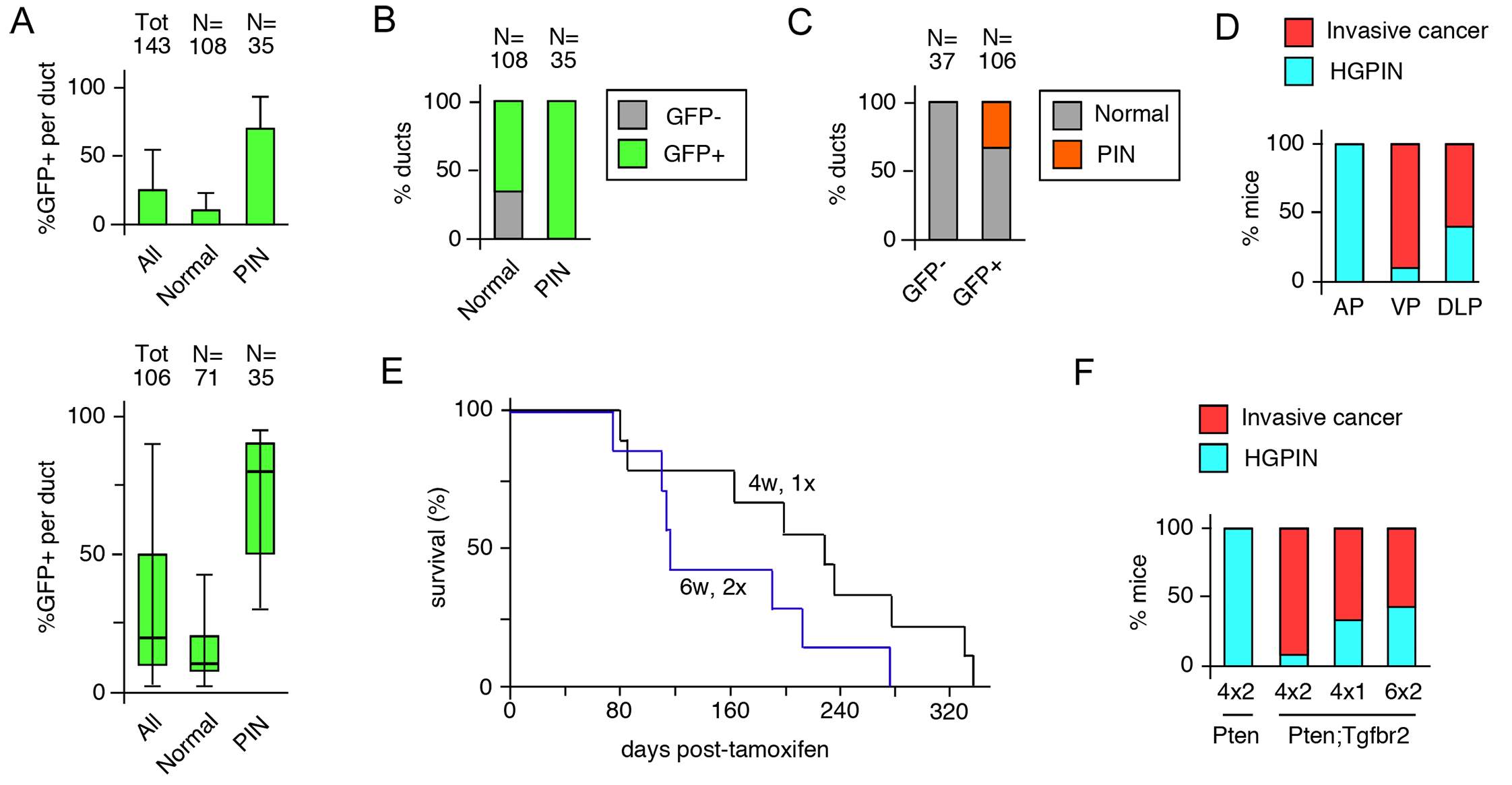

Supplement: S6 Fig — A) 143 ducts (selected randomly without first visualizing GFP staining) in mice 4–6 weeks after tamoxifen were scored for the proportion of cells that were GFP positive. The mean + sd is shown above for all ducts and separately for those with PIN or without phenotype. Below is the distribution of GFP positive cells per duct (excluding ducts without any GPF cells). Plotted as median, 25th and 75th percentiles (box) and 5th and 95th percentiles (whiskers). B) The proportion of normal and PIN ducts with GFP positive cells is shown. C) The proportion of GFP negative and GFP positive ducts with PIN is shown. D) The proportion of mice with either HGPIN or invasive cancer as the worst phenotype in each lobe (anterior [AP], ventral [VP] or dorsolateral [DLP] prostate) is shown, from the Pten;Tgfbr2 mice analyzed for survival in Fig 5D. E) Survival analysis for Krt8-CreERT2 Pten;Tgfbr2 mice treated either with one round of five days tamoxifen at four weeks (4w, 1x) or two rounds at six weeks (6w, 2x) of age is shown. F) Proportion of mice with either HGPIN or invasive cancer as the worst phenotype (in any lobe) at euthanasia for each treatment regimen (four weeks with one or two rounds: 4x1, 4x2 and six weeks with two rounds: 6x2), and for Pten single mutants is shown. (TIF) [file pgen.1007409.s006.tif]

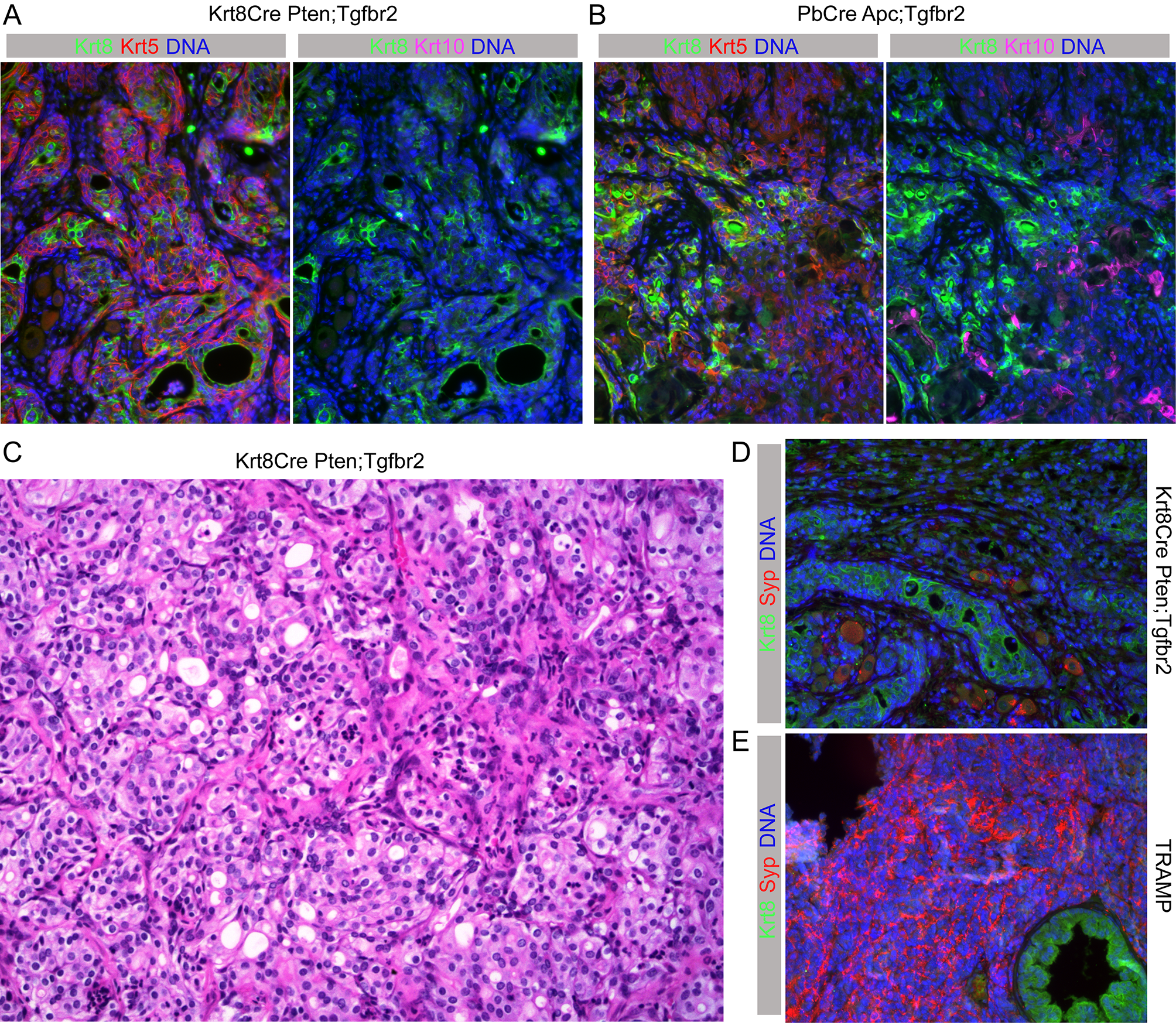

Supplement: S7 Fig — A) A Krt8Cre invasive double null tumor was stained for Krt5, Krt8 and Krt10 to examine squamous differentiation. B) An Apc;Tgfbr2 null tumor was analyzed for comparison–note the Krt10 signal in this tumor, which has squamous differentiation. C) High power (40x) image of H&E staining of an invasive Krt8Cre Pten;Tgfbr2 null tumor. D) A Krt8Cre invasive double null tumor was stained for Krt8 and Synaptophysin (Syp) to examine neuroendocrine differentiation. The region shown is representative of the highest Syp signal seen in these tumors. E) A TRAMP tumor is shown stained for Krt8 and Syp as a control for the neuroendocrine phenotype. (TIF) [file pgen.1007409.s007.tif]

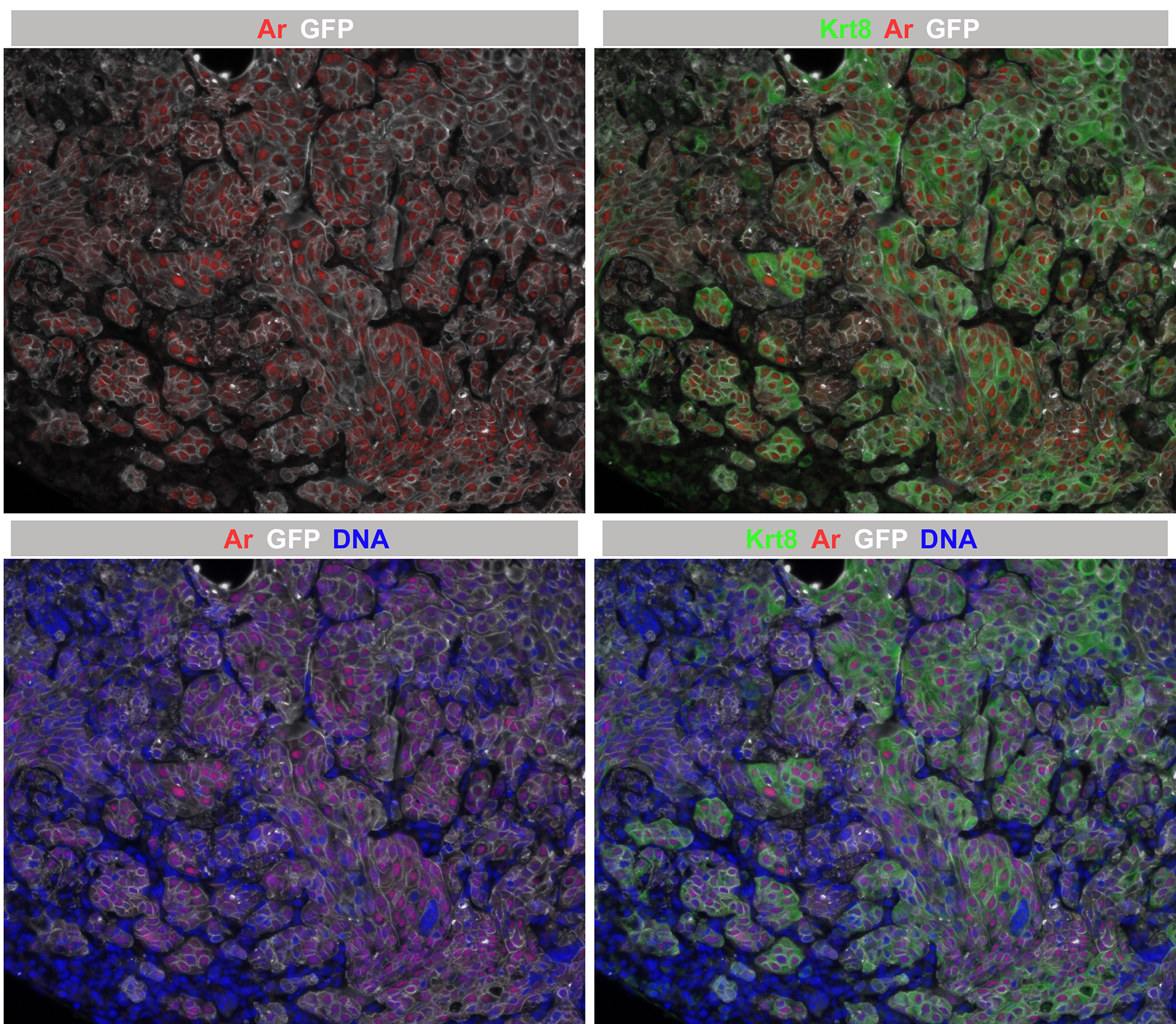

Supplement: S8 Fig — A lung tumor from a Krt8Cre invasive double null mouse was stained for Ar, Krt8 and GFP. Note that the majority of GFP cells are positive for Ar, with higher Ar signal generally associated with high Krt8 signal. This is consistent with the staining pattern seen in primary prostate tumors, consistent with lung tumors being prostate metastases. (TIF) [file pgen.1007409.s008.tif]
